# Validity, safety, usability, and user experience of virtual reality gamified home-based exercises in stroke

**DOI:** 10.1177/02692155251371435

**Published:** 2025-09-02

**Authors:** Hatem Lazem, David Harris, Abi Hall, Maedeh Mansoubi, Rodrigo Garcia Pontes, Carlos Bandeira de Mello Monteiro, Luciano Vieira de Araújo, Sarah E Lamb, Helen Dawes

**Affiliations:** 1 Medical School, Faculty of Health and Life Sciences, 151673 University of Exeter, Exeter, UK; 2 Basic Science department, Faculty of Physical Therapy, 63526 Cairo University, Cairo, Egypt; 3 School of Public Health and Sport Science, Faculty of Health and Life Sciences, 151673 University of Exeter, Exeter, UK; 4 School of Arts, Sciences and Humanities, 28133 University of São Paulo, São Paulo, SP, Brazil

**Keywords:** Stroke, telerehabilitation, virtual reality, games, validity, usability, qualitative

## Abstract

**Objective:**

This study adopted a novel approach to exploring the content validity, safety, usability, and user experiences of different games for telerehabilitation purposes from the perspective of physiotherapists and stroke survivors.

**Design:**

A cross-sectional content validity and usability study.

**Setting:**

Lab and online.

**Participants:**

23 participants were recruited; 11 neuro-physiotherapists and 12 chronic stroke survivors.

**Outcomes:**

Content validity and safety were assessed using a bespoke state evaluation questionnaire. The usability was evaluated using the system usability scale (SUS) and user experience questionnaire (UEQ). House of Quality analysis was conducted to identify the priority aspects for improvement.

**Results:**

Physiotherapists perceived the usability of the games as good to excellent for three games, median SUS = 80%, and poor for two games SUS < 68%. Three games had a mean average content validity index (CVI) > 0.8, and all games were safe to be administrated at home; mean CVI-safety item = 0.90. Stroke survivors with Fugl-Meyer Assessment of Upper Extremity function mean (SD) = 41(19.4), and mild to moderate spasticity perceived usability as very good to excellent for four games (median SUS = 85%). UEQ scale showed good to excellent acceptance among most of the games. House of Quality analysis revealed that clear instructions, avatar quality, motivational exercise scenarios, and clinical assessment tools are important criteria that should be considered throughout the development.

**Conclusion:**

This study demonstrated the value of exploring patient and physiotherapist perspectives for better telerehabilitation interventions co-development. Clinical trials should be conducted after further refinement of the games to investigate their feasibility and potential efficacy as a telerehabilitation tool for arm and balance training.

## Introduction

Globally, stroke is the second leading cause of death and the third leading cause of disability.^1^ There is an increase in its incidence due to the aging of the population.^2^ Stroke can lead to impairments in motor and sensory functions, speech, cognitive ability, and psychosocial difficulties.^3^ The functional decline experienced by people with stroke is commonly associated with increased dependence during daily activities and ultimately affects motivation levels, self-efficacy, and quality of life.^4^

Rehabilitation and physiotherapy remain a critical component in post-stroke management.^5^ A key factor in rehabilitation and relearning of motor skills post-stroke is the need for repetitive practice of functional tasks.^6^ Growing evidence suggests that a higher intensity of 1–2 hours of physiotherapy sessions per day is associated with greater improvement in daily activities for people with stroke,^7^ However, there is a shortage of physiotherapists in the United Kingdom.^8^

One means to support long-term and targeted rehabilitation is through telerehabilitation which utilizes technology for stroke survivors to work with therapists remotely and provide virtual access to rehabilitation services.^9^ Through telerehabilitation, healthcare professionals can monitor people with stroke remotely, and stroke survivors can access a greater volume of motor skill repetition and guided instruction. This can improve the patient's ability to perform home exercises and reduce clinic attendance.^10^ The pandemic COVID 19 accelerated the development of telerehabilitation tools.^11^

Telerehabilitation can incorporate various technologies such as virtual reality, augmented reality, mixed reality, mobile applications, and videoconferencing.^12,13^ In the virtual environment, including the gamified features, the patient receives visual feedback, which can be displayed using a head-mounted device, or flat screen.^14^ Virtual reality has been found to have an impact on the neuroplasticity of the brain,^15^ as well as enhancing performance in goal-oriented tasks and problem-solving capacity among the stroke population.^16^

Here, building on a previous review of the literature,^13^ feedback from users was explored during the early stages of development, to tailor the gamified exercises^17,18^ based on the expert physiotherapists’ recommendations of the exercises’ suitability, along with people with stroke abilities and preferences, to inform a person-based stroke telerehabilitation platform. The main objectives of this study were to determine the extent to which expert physiotherapists and stroke survivors reported the content validity, safety, usability, and user experience of five non-immersive virtual reality home-based exercise platforms.

## Methods

Ethical Approval was obtained under the clinical rehabilitation testing protocol (22-05-04-B-02) version 4, approved by the Research Ethics Committee at the University of Exeter.

A purposeful sample of physiotherapists with a minimum of 5 years’ experience in stroke rehabilitation with or without experience in telerehabilitation, and people with chronic stroke having problems in their upper limb function and balance abilities were recruited between September 2023 and February 2024. We circulated the Participant Information Sheet through social media, research groups (the University of Exeter research groups, personal connections with physiotherapists), and stroke supporting groups (Stroke Association UK, University of Exeter research groups, Exeter stroke peer supporting group, Action for Rehabilitation from Neurological injury group) to advertise the study.

Eligibility criteria for stroke survivors were: Chronic stroke ≥ 6 months from the initial stroke event; Age 18 years and above; Having mild to moderate spasticity of the shoulder and elbow joints assessed by Modified Ashworth Scale ≤ 2; Upper limb motor impairment was measured with Fugl-Meyer Assessment Upper Extremity ≤ 60; Self-reported balance problems; Sufficient cognitive ability to understand the instructions of the games was checked through by talking with them, and/or their carers in the online meeting or a phone call during the screening and recruitment stages. We recruited people with stroke able to adhere to two-phase instructions without explicit cognitive assessments in line with the practical needs of the intervention, as it depends on the participants’ capacity to understand and adhere to game instructions.

We excluded stroke survivors if they had: severe shoulder and /or elbow joint spasticity (Modified Ashworth Scale 3 or 4) and severe upper limb contractures (unable to passively extend their elbow joint reported by the physiotherapist during the baseline assessment). Eligible Participants were given 1 week to consider the information in the participant information sheet and ask any relevant questions before signing the informed consent.

The non-immersive virtual reality games used in this study have been developed by the School of Arts, Sciences, and Humanities of the University of São Paulo and previously used with non-stroke populations^17,18^ (Supplementary document 1). The games aim to encourage the unilateral and bilateral movements of the upper limbs motivating the participant to practice a higher number of upper limb reaching activities combined with somatosensory stimulation. The games also provide immediate and delayed summary sensory feedback (visual – hit and miss feedback; auditory – anticipatory and delay error), to support motor learning. The games include various motor and cognitive tasks designed to enhance problem-solving skills, memory, and visual-spatial reasoning abilities.

Data collection was conducted online for the physiotherapists individually via Zoom meetings. We started with familiarization with the intervention platforms and navigation through the options included in the platforms (30 min) and collection of demographic data, followed by a 10-min rest. Then, we shared the instruction sheet (Supplementary Document 1) about how to use the games and asked the physiotherapists to explore the platforms and practice exercise scenarios from sitting and standing positions (15 min for each game with 5 min of rest in between the games). After that, we asked the physiotherapists to fill out the content validity questionnaire and the system usability scale (SUS).^19^

Data collection for stroke survivors was conducted in-person at the virtual reality laboratory at the University of Exeter to ensure their safety while doing the exercises. We started with a pre-session assessment in which baseline demographic and motor performance data were recorded including upper limb function using Fugl-Meyer Assessment Upper Extremity,^20^ balance confidence using Activities-specific balance confidence scale,^21^ and spasticity assessed by Modified Ashworth Scale. Then, we explained the details of the games to the participants including the purpose of each game, and familiarized participants with the platform and all the games according to the instruction sheet (Supplementary Document 1) and no data were recorded. The participant was asked to sit or stand in front of the screen according to their balance abilities. They tried five games progressing through different levels of difficulty according to their abilities (15 min for each game with 5 min of rest in between the games). For the arm exercises, all the participants used either their affected arm or both arms, so the less affected arm just assisted the affected arm. For the balance exercises, all the participants used both of their arms. Games were presented in the same sequence to all the participants. After each game, people with stroke were asked to fill out three questionnaires (SUS, post-game satisfaction questionnaire, user experience questionnaire (UEQ)).

## Data collection and analysis

The content validity questionnaire was a bespoke state evaluation questionnaire that was designed by the experts in the research team (Supplementary Document 2), guided by examples from previous studies to assess the degree to which elements of the exercises are relevant and representative of the rehabilitation purpose.^22,23^ Based on the guidelines of Salkind (2013), at least two subject matter experts rate the content to establish validity.^24^

The usability of the games was assessed using the SUS.^19^ The scores ranged from 0 to 100%, where a higher score means better usability, with usability acceptance threshold of 68%.^19^ Nielsen reports that three to five users can identify 85% of relevant usability problems.^25^

A UEQ was used with people with stroke to evaluate each game against 26 items that were grouped into six scales: attractiveness, perspicuity, efficiency, dependability, stimulation, and novelty using a 7-point Likert scale ranging from −3 (fully agree with a negative term) to +3 (fully agree with a positive term).^26^ The average score has been calculated to measure the degree of agreement between the participants for each scale, then compared with the results of a benchmark data set from 468 studies examining different products and involving a total of 21,175 participants.^26^

The House of Quality is a tool designed to facilitate the correlation of people with stroke requirements with corresponding technical parameters enabling the identification of key aspects for enhancement.^27^ This structured approach aids in steering the focus of improvement efforts towards critical elements and optimizing the intervention adaptation processes in a cost-effective and time-efficient manner.^28^ The house of quality matrix consisted of users’ expectations score, satisfaction evaluation score, technical characteristics, and Interrelationship matrix^28^ (Supplementary Document 2). To understand the experiences of all users following their use of the games, we added open-ended questions at the end of the content validity questionnaire to ask physiotherapists about the suitability, safety, and applicability of each game's exercises as a stroke telerehabilitation tool, as well as patient satisfaction survey to ask about their feedback for each game (if any). We analysed these data using narrative synthesis.

$$\begin{array}{rl} & \overset{10\;criteria}{\underset{i=1}{\sum }}\;[user\;expectation\;weight_{i}\\ & \;\times \;interrelationship\;index\;\times \;(5-evaluation\;rating_{i})] \end{array}$$

## Results

Twenty-three participants (11 physiotherapists and 12 people with stroke) were recruited and participated in this study (see Table 1).

**Table 1.** Participants’ characteristics. All values are in median (Interquartile range), and n is the number of participants. FMA-UE (Fugl-Meyer Assessment - Upper Extremity), ABC (Activities-Specific Balance Confidence Scale), and MAS (Modified Ashworth Scale).

Physiotherapists	
N	11
Years of experience	
Median (minimum, maximum)	10 (5 - 20)
Country distribution	United Kingdom: 4 (37%)
n (%)	Egypt: 2 (18%)
	Indonesia: 2 (18%)
	Saudi Arabia: 2 (18%)
	China: 1 (9%)
People with stroke	
N	12
Age	61(31.50)
Gender	Females: 4 (33.3%)
n (%)	Males: 8 (66.7%)
Affected side	Left side: 10 (83.3%)
n (%)	Right side: 2 (16.7%)
Stroke sub-type	Haemorrhagic: 3 (25%)
n (%)	ischemic: 8 (66.7%)
	unknown: 1(8.3%)
Time from stroke (months)	46(72.38)
FMA-UE	34.5(39.5)
ABC	80(29.92)
MAS	1.5(1)
Multimorbid conditions	Hypertension: 5 (41.67)
n (%)	Chronic low back pain: 2 (16.67) Knee Osteoarthritis: 2 (16.67)
	Rotator cuff injury: 2 (16.67)
	Knee replacement: 1 (8.3%)
	Intramedullary nail fixation: 1 (8.3%)
	Controlled epilepsy: 1 (8.3%)
	Gout: 1 (8.3%)
	COPD: 1 (8.3%)
Favourite game	Move Hero Game: 5 (41.67%)
n (%)	Basketball Game: 4 (33.33%)
	Puzzle Game: 2 (16.67)
	Flowers Game: 1 (8.3%)
Least favourite game (absolute number /total number)	Magic Pattern Game: 9 (75%)
	Puzzle Game: 2 (16.67)
n (%)	Basketball Game: 1 (8.3%)

**Figure 1.**
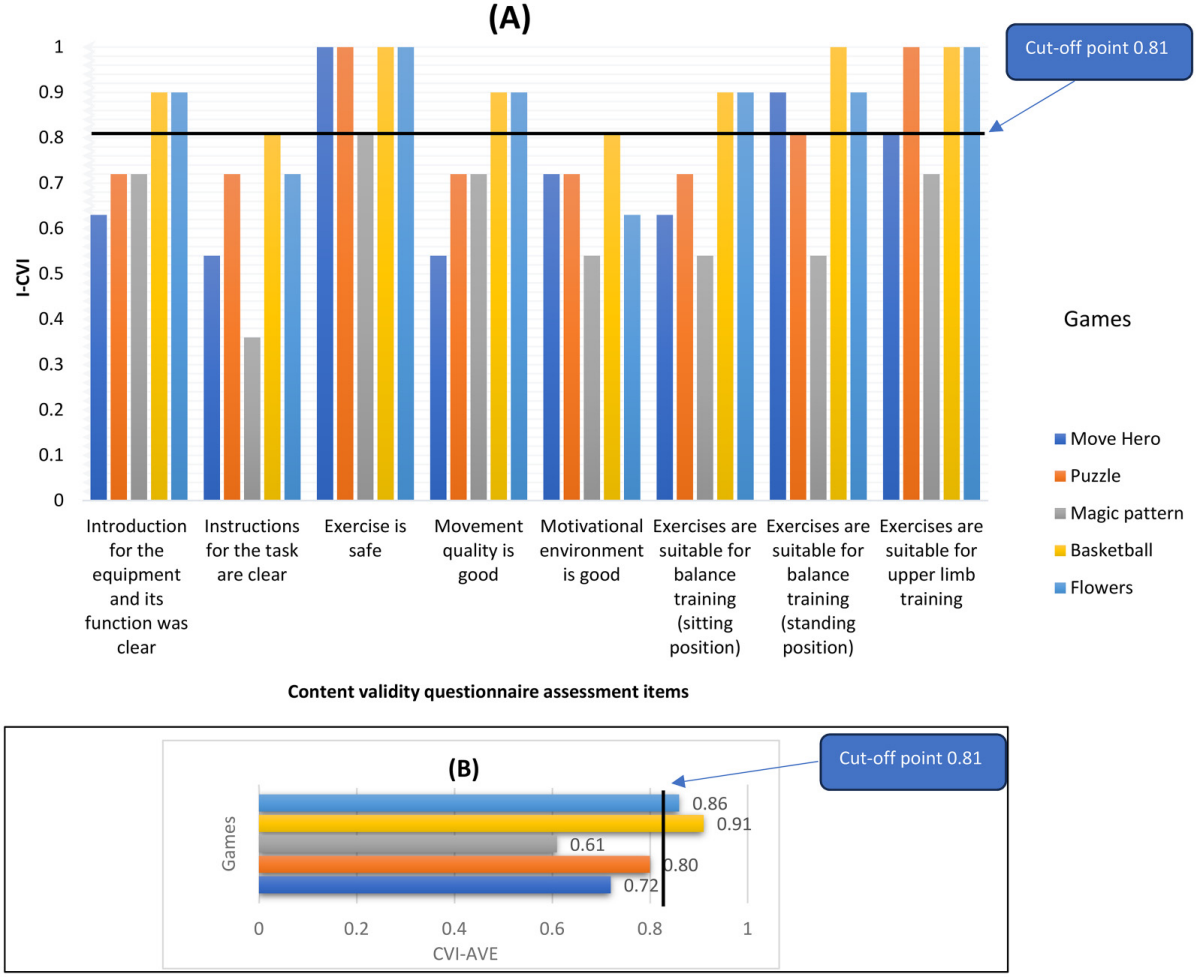
(A) I- CVI (Item-Content Validity Index), and (B) CVI-AVE (Average-Content Validity index) of the games based on the content validity questionnaire analysis.

All physiotherapists agreed that all exercises would be safe for people with stroke to practice as part of their home rehabilitation routine with an average safety Item-Content Validity Index (I-CVI) = 0.90. All the games except Magic Pattern were reported to be suitable for upper limb training and balance training from a standing position with I-CVI > 0.81. Basketball and Flowers games were reported to be suitable for balance training from a sitting position; I-CVI = 0.90. Regarding motivation, only the basketball game showed good potential to offer a motivational environment for people with stroke. Both Basketball and Flowers games provided suitable exercises that can achieve good and acceptable movement quality. Expert physiotherapists agreed that only Puzzle, Basketball, and Flowers games are suitable for telerehabilitation; Content Validity Index-Average (CVI-AVE) ≥ 0.80 (Figure 1). (Supplementary document 2)

Based on the open-ended question responses from the physiotherapists, we can categorize their feedback into six main categories: game instructions, avatar quality, exercise safety, movement quality, performance feedback, and exercise scenarios. All the included games lack clear instructions for how to start the games, set up the needed equipment to perform the exercise accurately, and lack instructions for the physiotherapists on how to tailor the exercise program. Two games (Move Hero and Pattern Magic) showed a less clear avatar that can easily be affected by the lighting of the room and the position of the camera while the other three games showed good quality regarding the accuracy of the graphics as the camera can detect the whole pictures of the body not just an avatar. All experts recommended that all exercises included in these five games can be safe to be administrated as part of the upper limb and balance training as a telerehabilitation tool as people with stroke can do these exercises from sitting or standing position with many levels of difficulties according to their abilities. Most of the included games need some adaptation for some parameters to avoid patient frustration from doing the exercises due to the inappropriate exercise parameters.

Despite all the games giving sensory feedback in the form of auditory and visual feedback, there is a lack of tactile stimulation, which is one of the important sensory feedback items during the recovery process. Difficulty levels, progression of exercises, and the exercises’ aim are the main three items that should be considered for tailoring an effective home-based exercise program (For further details, check Supplementary Document 2).

**Figure 2.**
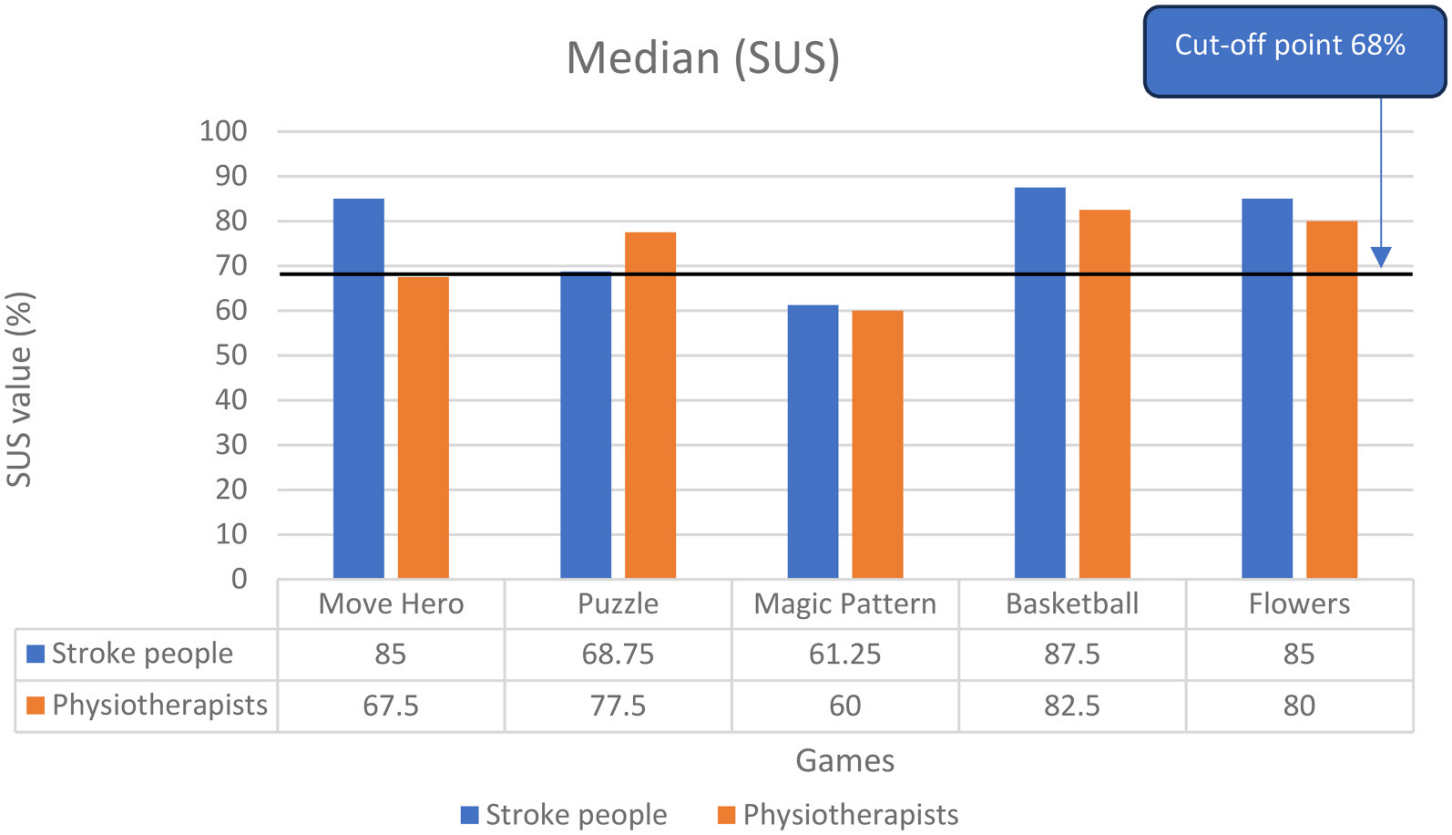
Median values of the system usability scale (SUS) rating among both physiotherapists and people with stroke for the five games (Move Hero, Puzzle, Magic Pattern, Basketball, Flowers).

In terms of the intervention usability, the median usability perception scores, as assessed by the SUS for the More Hero, Puzzle, Magic Pattern, Basketball, and Flowers games were 67.50%, 77.50%, 60%, 82.50%, and 80% among physiotherapists and were 85%, 68.75%, 61.25%, 87.50%, and 85% among people with stroke, respectively. Only three games showed a good system usability agreement among all the participants (acceptable cut-off point for good system usability; system usability score > 68%)^29^ (Figure 2). All games’ item scores were close to the best usability item analysis scores, except the Magic Pattern game. These games were rated as easy to use, well-integrated, required minimal support for administration, and could be confidently used with the stroke population (Supplementary Document 2).

UEQ analysis was used to calculate the mean values of the total score. Mean values between −0.80 and 0.80 represent a neutral evaluation, values >0.80 correspond to a positive evaluation, and values < -0.80 refer to a negative evaluation.^30^ Standard deviation of UEQ scales Schrepp (2023)^30^ showed that stroke survivors had a low level of agreement about their positive or neutral experience after using the games, except for Move Hero, Basketball, and Flowers, where there was moderate to high agreement in most scales (Supplementary Document 2).

**Figure 3.**
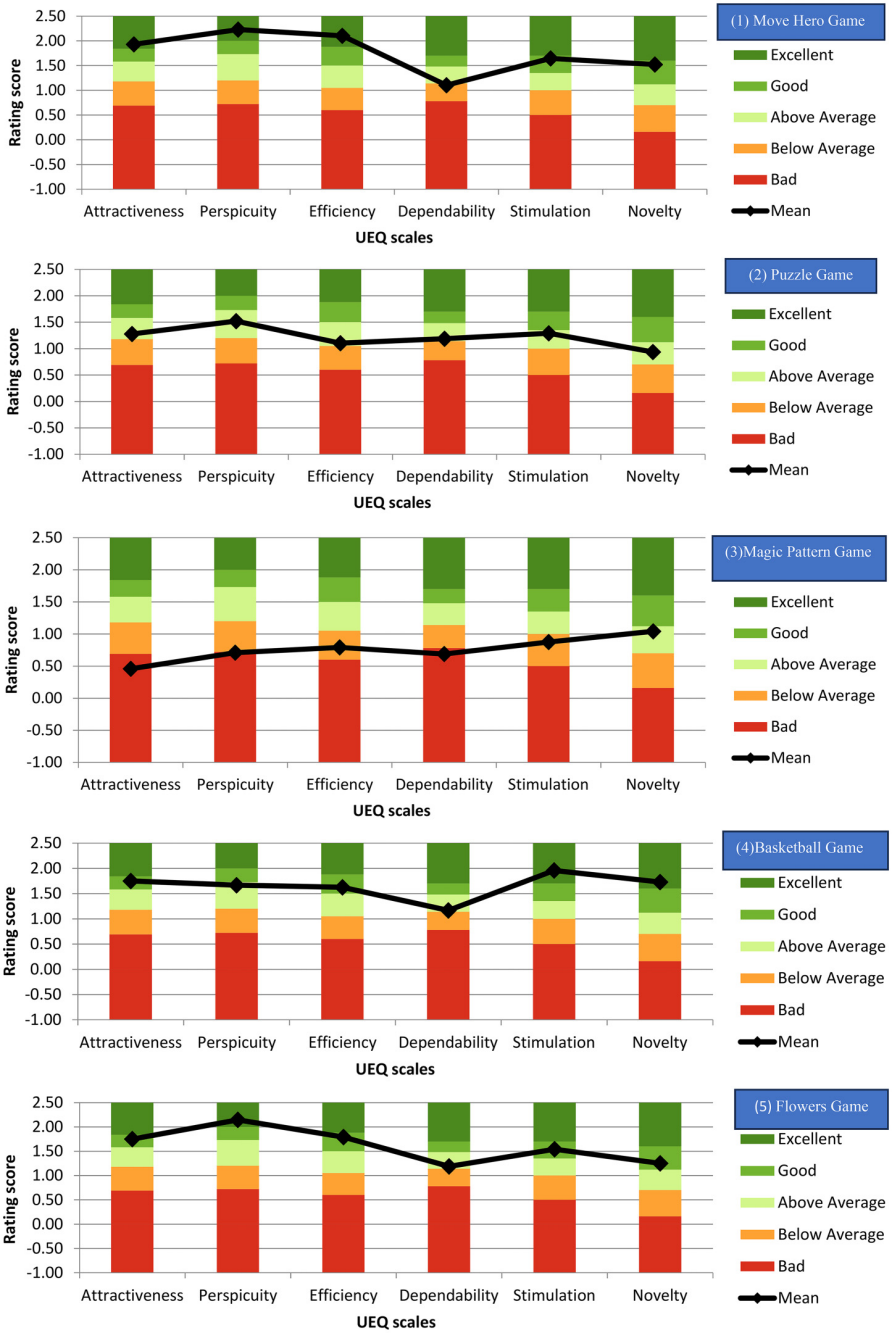
Results of the user experience questionnaire (UEQ) aggregated into six scales: attractiveness, perspicuity, dependability, stimulation, and novelty. Comparison of stroke survivors’ rating evaluation results between the five games and the user experience questionnaire benchmark.

The measured scale means were set in relation to existing values from a benchmark data set. This data set contains data from 21175 persons from 468 studies (Supplementary Document 2). Compared to the benchmark values, mean rating score values of Move Hero, Basketball, and Flowers games showed a good to excellent score ranges regarding the patient experience among attractiveness, efficiency, stimulation, and novelty scales but not for the dependability and the perspicuity (Move Hero: below average dependency M (SD) = 1.10(0.7), Basketball: above average dependability and the perspicuity M (SD) = 1.16(0.97), 1.66(1.04) respectively, Flowers: below average dependency M(SD) = 1.18(0.96)) (Figure 3).

All the scales were above average for the puzzle game, and below average or bad for the Magic Pattern Game except for the novelty scale; above average M(SD) = 1.04(1.02). A high score on the perspicuity measure indicates that most of the games were comprehensible and user-friendly for people with stroke (Figure 3).

The distribution of the responses for the 26 UEQ items was analysed, according to their score on the Likert scale (Supplementary Document 2). It was shown that there was a positive or neutral evaluation ≥ 80% by the stroke survivors with the total number of items being 25, 18, 8, 23, and 24 for the Move Hero, Puzzle, Magic Pattern, Basketball, and Flowers games, respectively.

According to the House of Quality analysis, all 10 criteria were important to be considered in any telerehabilitation non-immersive virtual reality games (median rating ≥ 4). The most satisfactory criteria were ‘adequate difficulty’ and ‘improve functional abilities (efficiency)’, while the least satisfactory criteria were ‘score tracking’ and ‘easy to use criteria’ (Supplementary Document 2).

**Figure 4.**
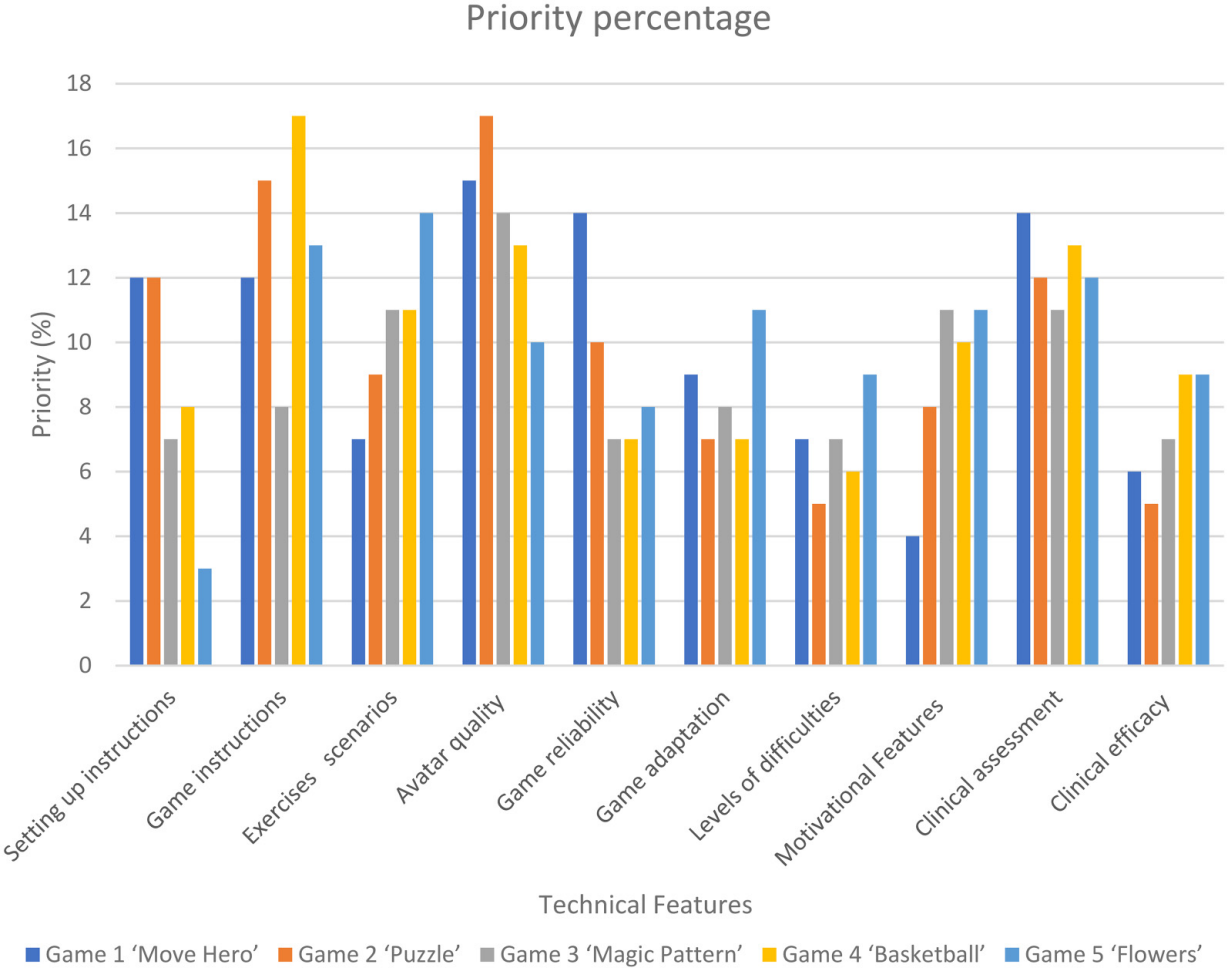
Priority percentages (normalized priority weights) for individual technical characteristics.

Based on the priority percentage, there was a degree of variability across the games; however, the top five priority needs for improvement across most of the games were ‘Game instructions’, the inclusion of ‘clinical functional assessment’, ‘avatar quality’, ‘motivational features’, and ‘exercise scenarios (Figure 4).

## Discussion

The validation results suggested that the gamified exercises provide safe motivational environments that enable people with stroke to engage in exercise practice with a significant number of repetitions, minimal compensations, and a high level of adherence. Both motivation and engagement are key elements of an effective stroke rehabilitation plan, additionally enhancing people with stroke autonomy in daily activities.^31^

Nevertheless, a significant proportion of the games have a shortage of explicit guidelines about their use and a lack of information for physiotherapists on how to tailor the exercise program. This outcome is consistent with a study that found lack of knowledge among physiotherapists can hinder intervention adoption in practice.^32^

System usability results indicate a high level of system usefulness across four games. A notable disparity was seen in the rating scores of both Move Hero and Puzzle games between physiotherapists and people with stroke. According to physiotherapists, the Puzzle game is appropriate for stroke rehabilitation compared to the Move Hero game as the Puzzle game incorporates more cognitive demand in its tasks, which enhance problem-solving abilities, memory, visual and spatial reasoning skills, as well as functional motor tasks. In contrast, the Move Hero game primarily emphasizes more on the motor tasks with less cognitive elements. Conversely, people with stroke reported that the Move Hero game was more user-friendly compared to the Puzzle game, which posed challenges in terms of usability and score tracking according to the House of Quality analysis. The basketball game had a commendable level of usefulness as reported by both the physiotherapists and people with stroke. Nevertheless, it encompasses activities that require motor, visual, and spatial thinking abilities.

These findings indicate that integrating motor and cognitive tasks is significant in stroke rehabilitation, however, also highlight a need to ensure that tasks are comprehensible and effectively motivate people with stroke, enabling them to perceive progress in their abilities without experiencing frustration stemming from task complexity.^33^ Our findings align with a study that observed that not all gamified exercises possess the neurobiological responses associated with gamification, such as enhanced attention, visual-motor skills, and cognitive-motor efficiency.^34^ This lack of response may prevent the desired outcomes of the exercise gamification.^35^ Therefore, it is essential to consider patient feedback regarding engagement as a variable, as it has been proven to significantly influence therapeutic outcomes.^36^

Findings from UEQ analysis reveal a positive evaluation with high scores in the Basketball and Flowers games which contain competition factors against opponent avatars in their exercise scenario. Competitive exercises can improve the degree of motivation and enjoyment for the users.^37^ House of Quality analysis outcomes might be attributed to the inclusion of diverse exercises with varying complexity levels, which can render the exercises stimulating and encouraging for participants to engage in a significant number of exercise repetitions, hence impacting their adherence to the exercise program. Both manual and automatic difficulty modulation in games can enhance motivation and increase the intensity of upper limb rehabilitation exercises.^38^ Furthermore, the provision of a variety of games can offer people with stroke the opportunity to engage in exercises at home that align with their individual behavioural preferences. Pragmatic quality can be improved by incorporating a precise clinical assessment tool using computer-vision software, improving the perspicuity of the games.

However, we included People with stroke with different levels of arm and balance abilities; we didn’t observe any significant differences in the usability rating relative to their impairment level. This finding supports the adaptability and inclusivity of the games, making them suitable across different impairment levels in stroke telerehabilitation. This can be useful for physiotherapists who can use the same games and adapt the parameters to be personalized with different impairment levels among stroke populations.

Numerous virtual reality platforms have been utilized for stroke rehabilitation and telerehabilitation^12,13^ including specific and non-specific virtual reality exercises, but only a limited number of studies have assessed the usability and content validity of these platforms among people with stroke and physiotherapists before evaluating the feasibility and effectiveness of the intervention as a telerehabilitation tool.^28,39,40^ This can influence the clinical efficacy and the acceptance of the intervention to be used at home among the users.^33^

This study has some potential limitations. The sample of stroke participants was limited to those from the United Kingdom. This lack of diversity in terms of cultural acceptance of the intervention suggests that the intervention may not be widely accepted by a broader community. The lasting effect of participants’ motivation and involvement with the gamified exercises is undetermined, as our study only spanned a single session. The study has not examined potential technical challenges, such as software glitches, hardware failures, or network problems, which could impact the usability and efficacy of the intervention.

**Figure 5.**
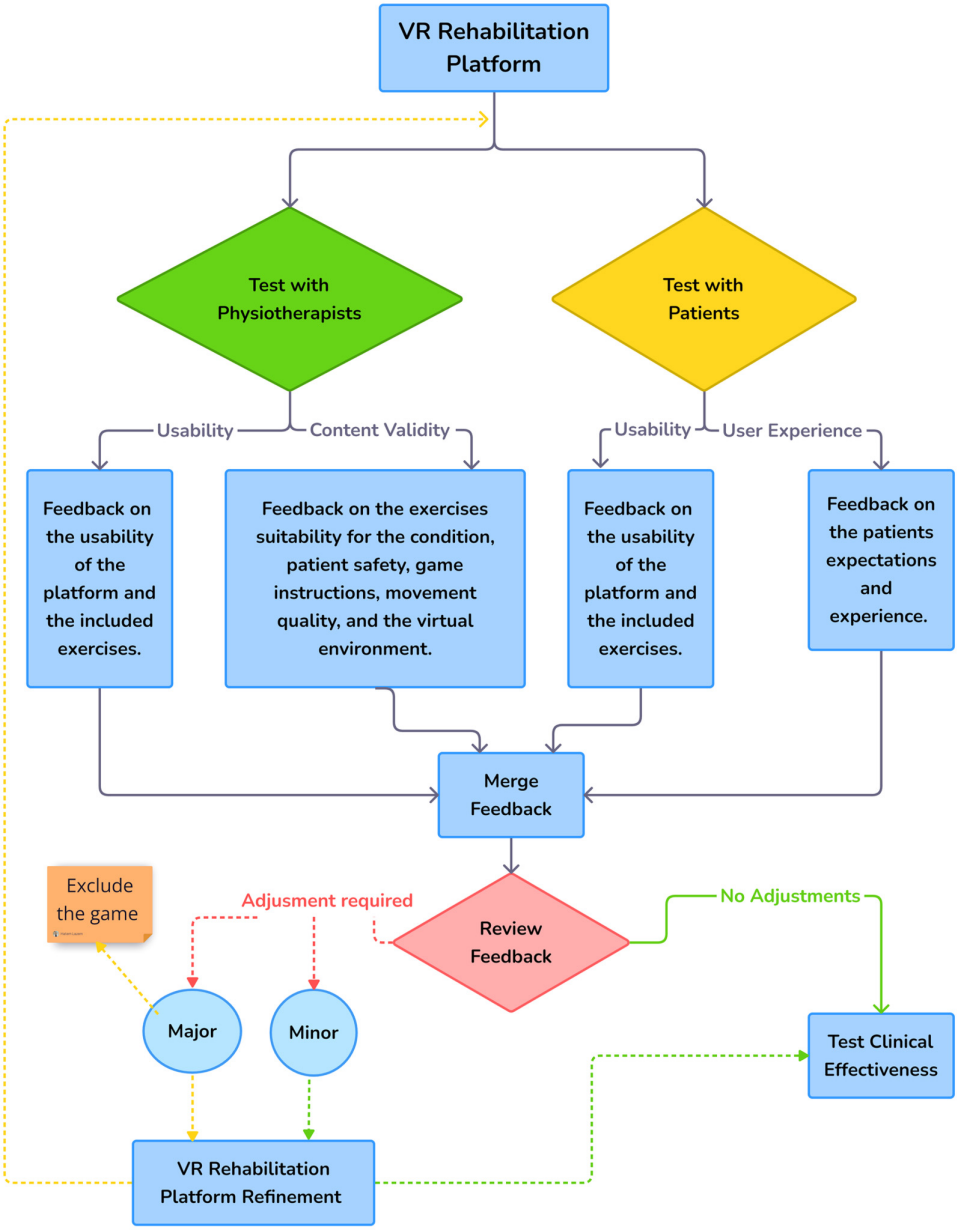
The figure illustrates a suggested sequence of steps that should be tested on any virtual reality rehabilitation platform prior to evaluating its clinical impact on any given condition. Based on our study, we recommend that researchers should start with testing the usability, content validity, and user experience among both target people with stroke and the physiotherapists, then combine all the feedback and decide if the virtual reality platform is ready for testing its feasibility and clinical effectiveness, or needs further refinement. This iterative process will ensure that the platform not only meets the needs of its users but also aligns with the therapeutic goals set by healthcare professionals.

Compared to the previous studies,^28,39,40^ our study is the first to combine and analyse the usability testing, content validity assessment, and user experience evaluation of virtual reality games as a home-based intervention among people with stroke and physiotherapists. This allows us to develop a comprehensive understanding of the intervention and identify domains for improvement before testing its clinical efficacy. We suggest using a conceptual map (Figure 5) to evaluate gamified virtual reality exercises before the deployment stage of development.

**Clinical messages**

- Physiotherapists and people with stroke were positive about using adapted virtual reality games to enhance motivation to practice home-based exercises.
- Physiotherapists can design a safe personalized telerehabilitation program for people with stroke.
- Using simple interfaces (webcam) can enhance the adoption of game therapy in motor rehabilitation.
- Assessing the validity and safety of any virtual reality intervention is crucial to the intervention's acceptance.

## Supplemental Material

sj-docx-1-cre-10.1177_02692155251371435 - Supplemental material for Validity, safety, usability, and user experience of virtual reality gamified home-based exercises in stroke Supplemental material, sj-docx-1-cre-10.1177_02692155251371435 for Validity, safety, usability, and user experience of virtual reality gamified home-based exercises in stroke by Hatem Lazem, David Harris, Abi Hall, Maedeh Mansoubi, Rodrigo Garcia Pontes, Carlos Bandeira de Mello Monteiro, Luciano Vieira de Araújo, Sarah E Lamb and Helen Dawes in Clinical Rehabilitation

sj-docx-2-cre-10.1177_02692155251371435 - Supplemental material for Validity, safety, usability, and user experience of virtual reality gamified home-based exercises in stroke Supplemental material, sj-docx-2-cre-10.1177_02692155251371435 for Validity, safety, usability, and user experience of virtual reality gamified home-based exercises in stroke by Hatem Lazem, David Harris, Abi Hall, Maedeh Mansoubi, Rodrigo Garcia Pontes, Carlos Bandeira de Mello Monteiro, Luciano Vieira de Araújo, Sarah E Lamb and Helen Dawes in Clinical Rehabilitation
