# Supplementary material for: Validity, safety, usability, and user experience of virtual reality gamified home-based exercises in stroke
Source: Clin Rehabil. 2025 Sep 2;39(11):1527–40. doi: 10.1177/02692155251371435 (PMC12521778; doi:10.1177/02692155251371435)
Supplement: sj-docx-1-cre-10.1177_02692155251371435 - Supplemental material for Validity, safety, usability, and user experience of virtual reality gamified home-based exercises in stroke [file sj-docx-1-cre-10.1177_02692155251371435.docx]

**Supplementary document 1**

**Non-immersive Virtual reality telerehabilitation Exercises (Games)**

**Setting up:**


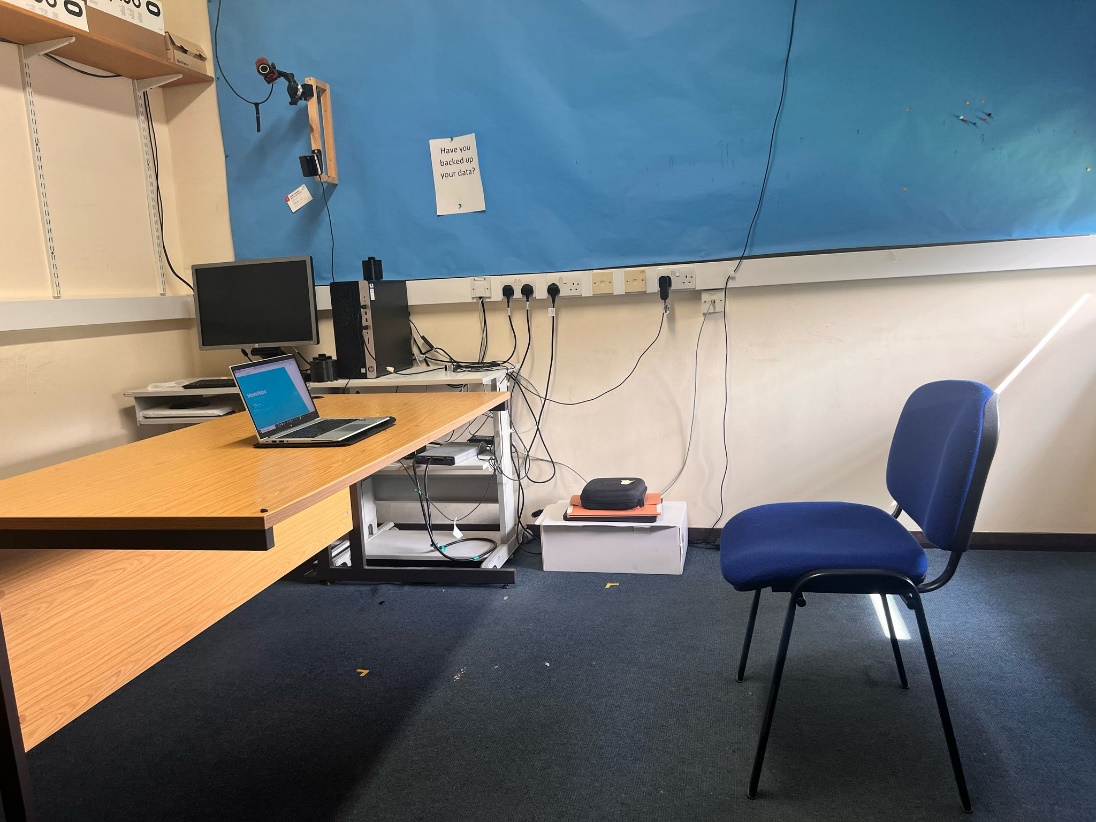


The chair that will be used by the participant (stroke survivor) if they will do the exercises from sitting position. We will remove the chair, and the participant will stand at the same position if they will practice the exercises from standing position.

Distance between the laptop and the position of the participant (1.5 meter)

The laptop that will be used during the experiment. exercise

**Fig 1: room setting up.**


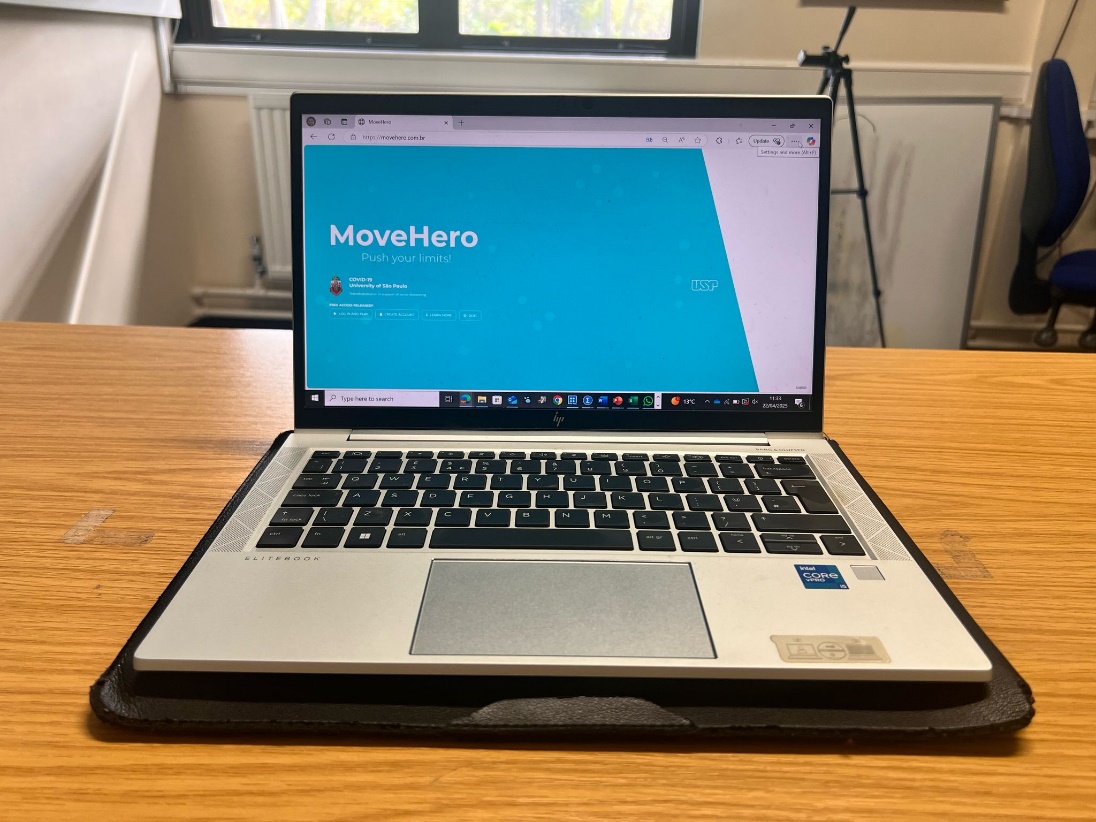


Laptop camera will be used to detect the participant body as a mirror avatar.

**Fig 2: laptop used to practice the exercises**

**Games Description**

**1-Move-Hero game:** this game aims to improve the unilateral and bilateral movements of the upper limbs in the form of motivating the participant to practice a higher number of upper limbs reaching activities combined with somatosensory stimulation. The game scenarios consist of a screen containing 4 circles in different positions reflecting a variety of range of movements required from the participants. There will be balls with different colors falling from the top of the screen and the participant should touch the circle once the ball is in this circle. The participant is positioned in front of a computer (in sitting or standing position according to their balance ability assessed by the researcher) and when the game starts the webcam captures the participant’s movements and a representation of the player appears on the computer screen as an avatar. The goal of the game is to intercept all falling spheres using upper limb wave movements from either sitting or standing positions at the exact moment the spheres reach their specific target at the bottom of the computer screen. The game presents four columns with fixed parallel targets allocated at two height levels (e.g., two on the left - targets A and B; two on the right – targets C and D). The game also provides somatosensory feedback (visual – hit and miss feedback; auditory – anticipatory and delay error, proprioceptive stimulation in the form of stimulating the joint proprioception) – if the individual reaches the spheres correctly, the game presents feedback with the spheres changing the color of the target to blue, with little stars around it (hit information). On the other hand, if the participant does not reach the spheres correctly, the spheres change color to red and the letter X appears inside the target together with auditory feedback. The participant will be asked to move his hand trying to catch the ball when touching the circles either using hand movement from sitting or standing positions. **Fig 3**


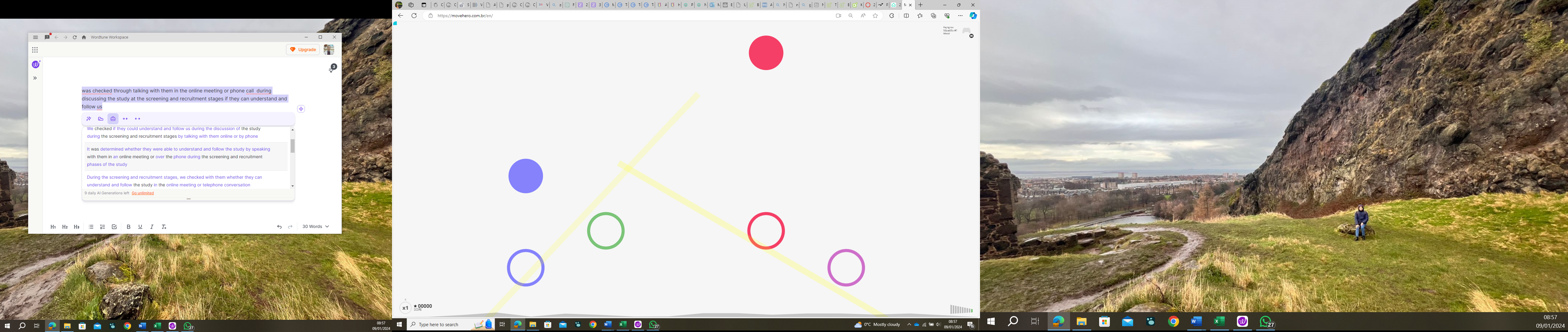


**Fig 3: Move Hero Game**

**Instructions about how to use the game:**

| **Move-Hero game** | |
| --- | --- |
| Step 1 | Please open this link: <https://movehero.com.br/> from google chrome browser. |
| Step 2:   - Click on create account. | 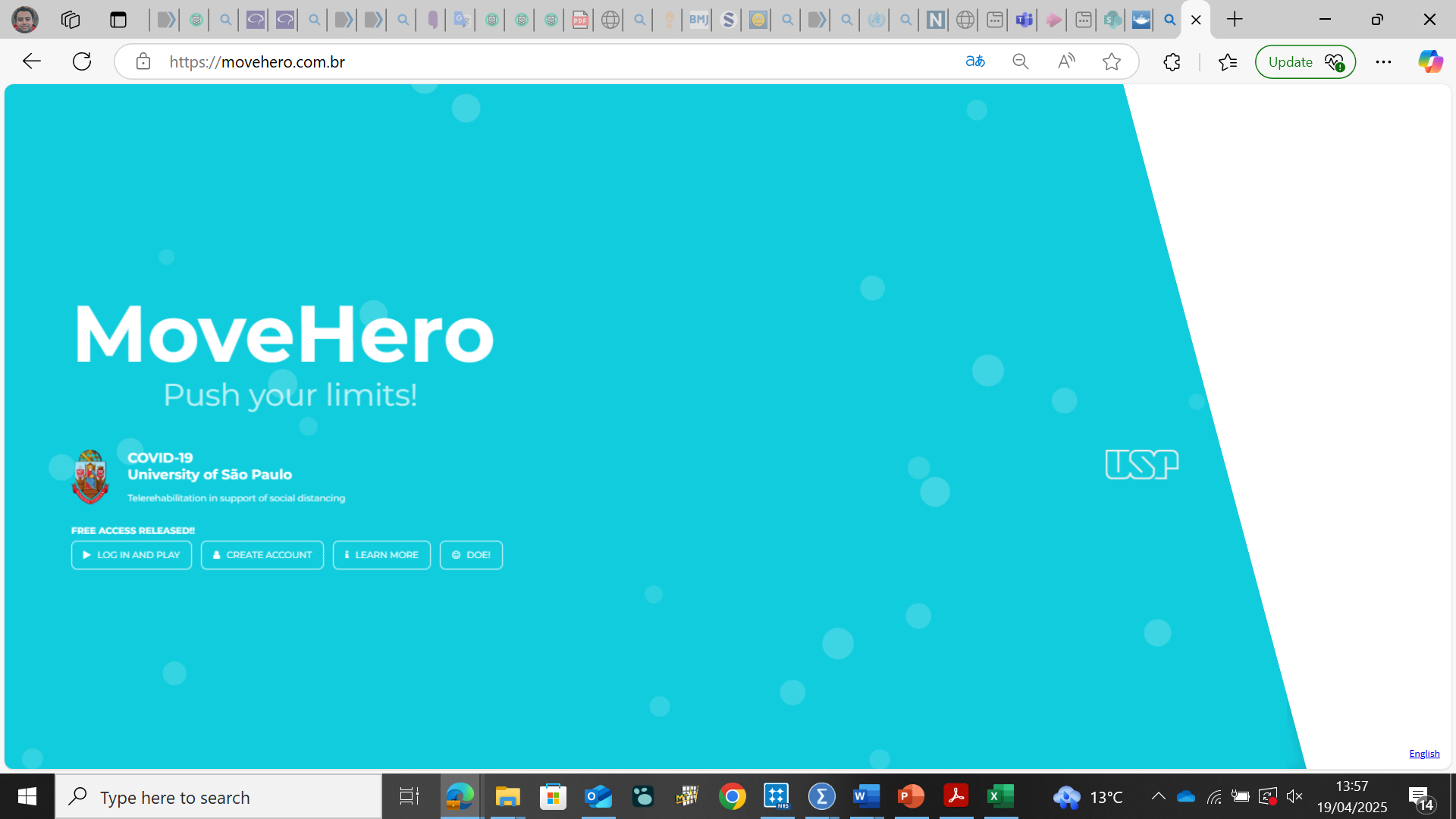  **Click here** |
| Step 3:   - After creating your account, click on play. - Then, you can choose your favourite song that you want to listen to while doing the exercises. - Then click on protocol, and choose between Fase 1 (easy level) to Fase 8 (difficult level). - For stroke survivors, the researcher will help then to choose the best level that is suitable for them (not too easy so there will be no challenges, and not too difficult). - **For the physiotherapist**, they have to try the different 8 levels to be able to judge their suitability as a home-based exercises for people with stroke. | 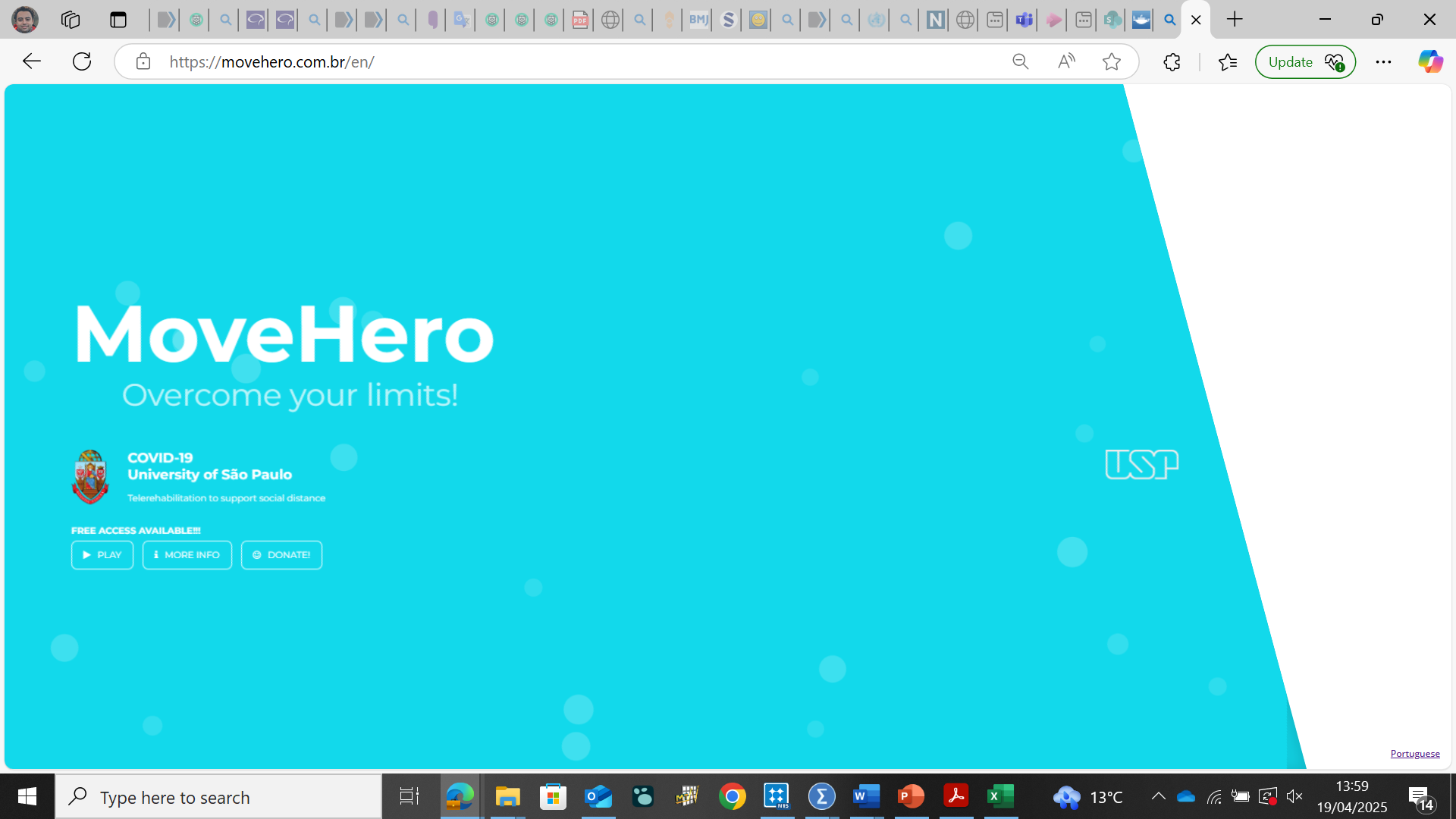  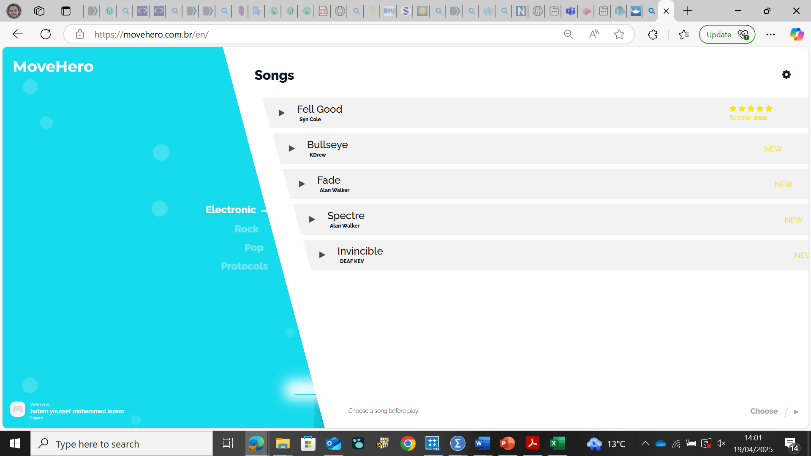  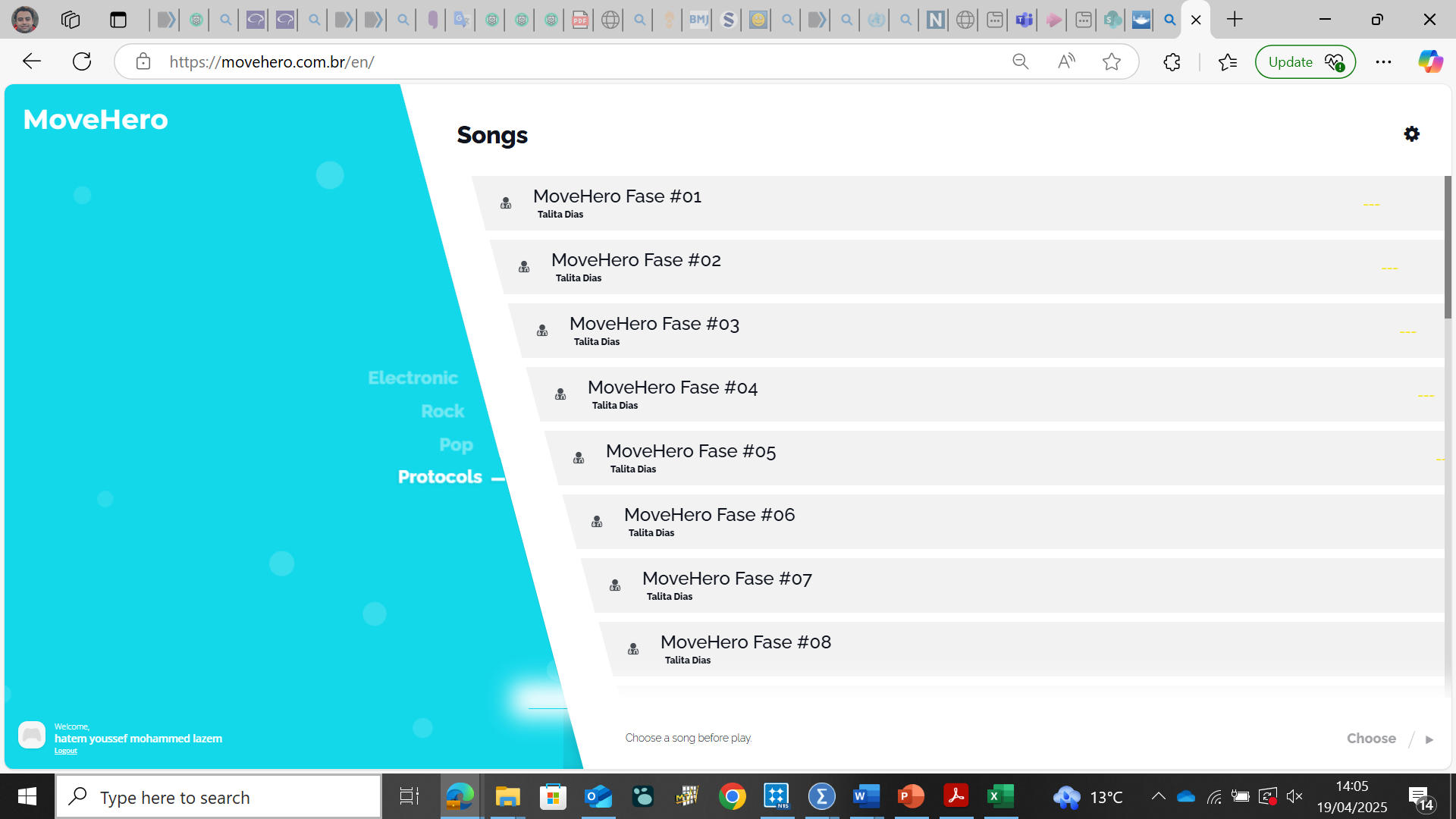 |
| - When you start the game, this page will appear on the screen. You will appear as a mirror avatar on the screen. Your head should be in the middle of the screen. You should be away from the screen with a distance about 1.5 meters. - **The task:** now you have to raise your affected arm trying to touch the circle once the falling balls reach the circle. If you can not raise your affected arm toward the targets, you can use tour sound arm to helm the affected arm. | 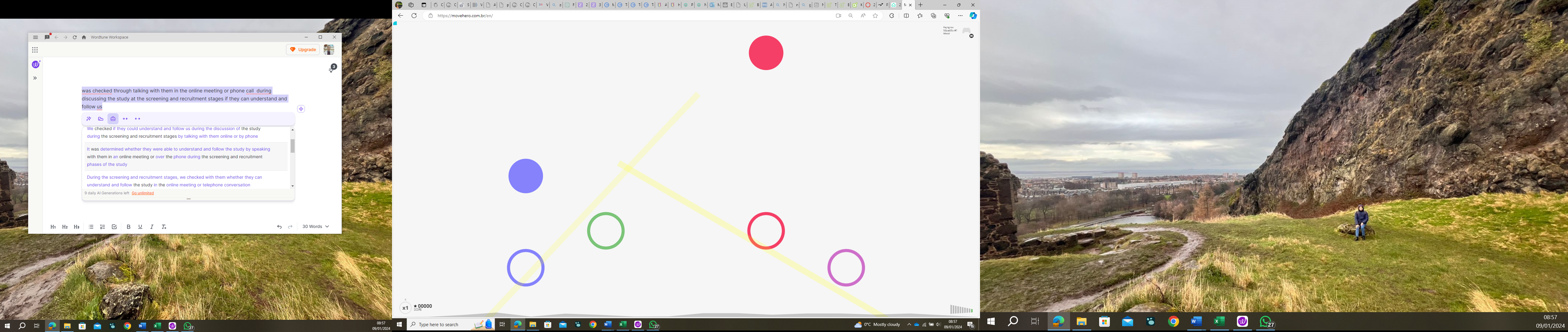 |
| - **Note** | If you experience any technical difficulties, please contact the research team. |

**2-Puzzle game:** this game aims to improve bilateral hand movements and cognitive abilities by providing sensory feedback to the participant and encouraging manipulation of the puzzle pieces by moving the whole upper limb and the hand to slide the 4 pieces of the puzzle into their correct positions in the centre of the screen to form the correct picture. We can manipulate the position of the 4 pieces of the puzzle and the target position according to the abilities of the participant; if the participant has hemineglect, we can put all of the tasks on the right side of the screen, and if the patient has a limited range of motion of the shoulder, we can put the task in the lower part of the screen and all of this modification can allow the participant to practice their exercises without many substitutions and with high degree of motivation without being upset from the complexity of the task. **Fig 4**


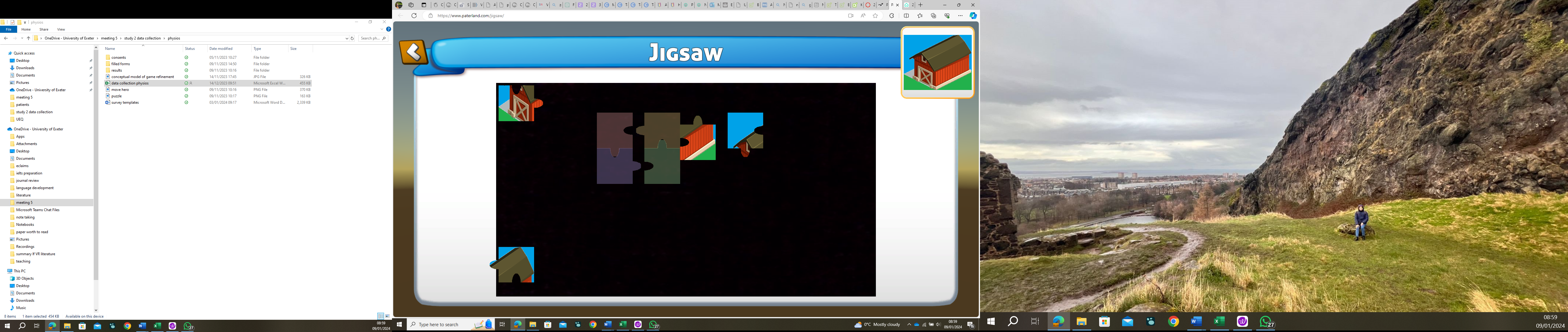


**Fig 4: Puzzle Game**

**Instructions about how to use the game:**

| **Puzzle game** | |
| --- | --- |
| Step 1 | **Click on the puzzle game link. https://www.paterland.com/jigsaw/** |
| Step 2:   - In the first screen, click in the green Start button, then, you will need to input your identifier in the blue box and the research password (REHABEXETER) in the orange box. Finally, click in the green button to proceed to the next screen. | **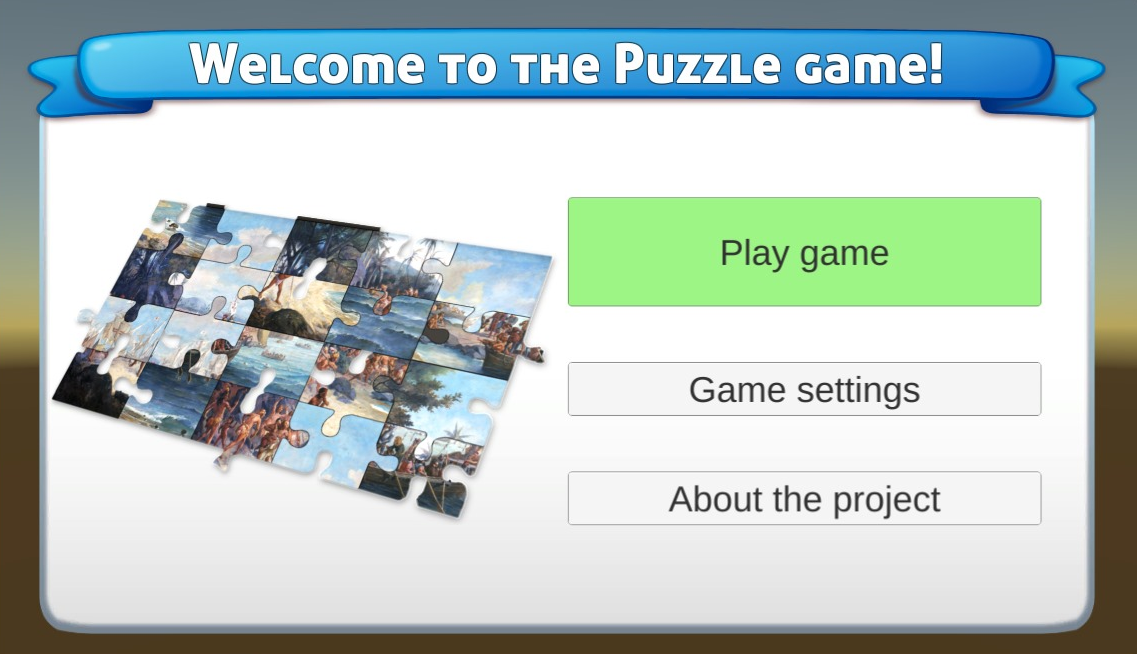**  **Choose this**  **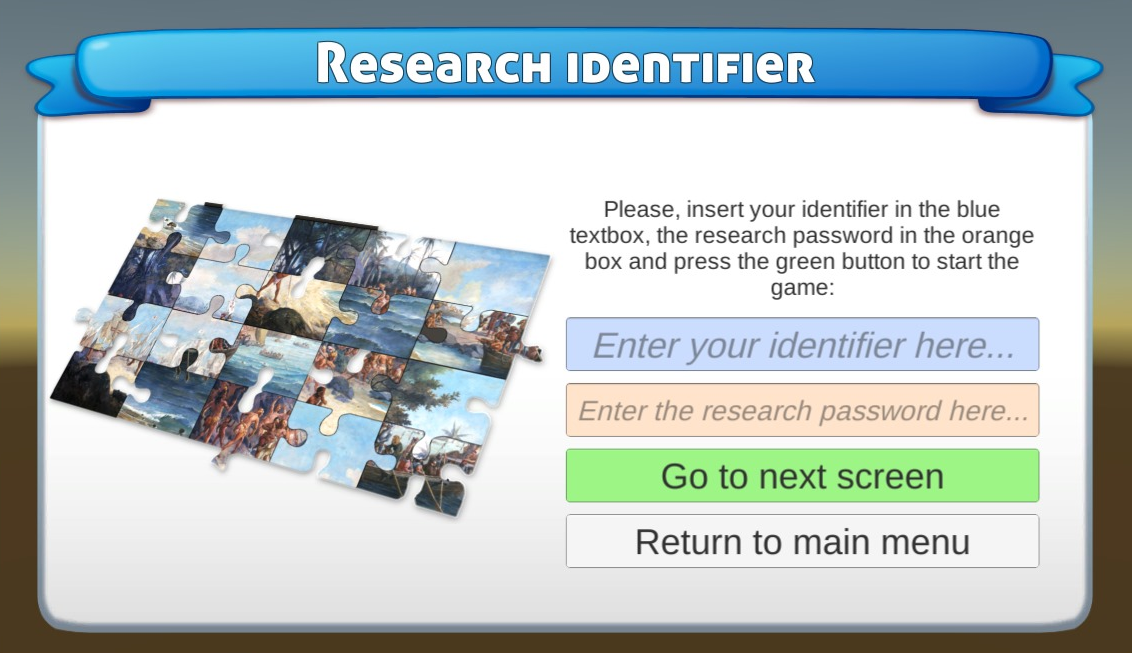** |
| - When you start the game, this page will appear on the screen. You will be able to see your picture on the screen. Your head should be in the middle of the screen. You should be away from the screen with a distance about 1.5 meters. - **The task:** now you move both of your arms trying to put the 4 pieces of the puzzle in their correct position on the middle of the screen. You can do it from sitting or standing positions based on your physiotherapist recommendations. - **For the physiotherapists**, you will have to try doing these exercises from both sitting and standing position to be able to assess their usability as a telerehabilitation exercises for people with stroke. | **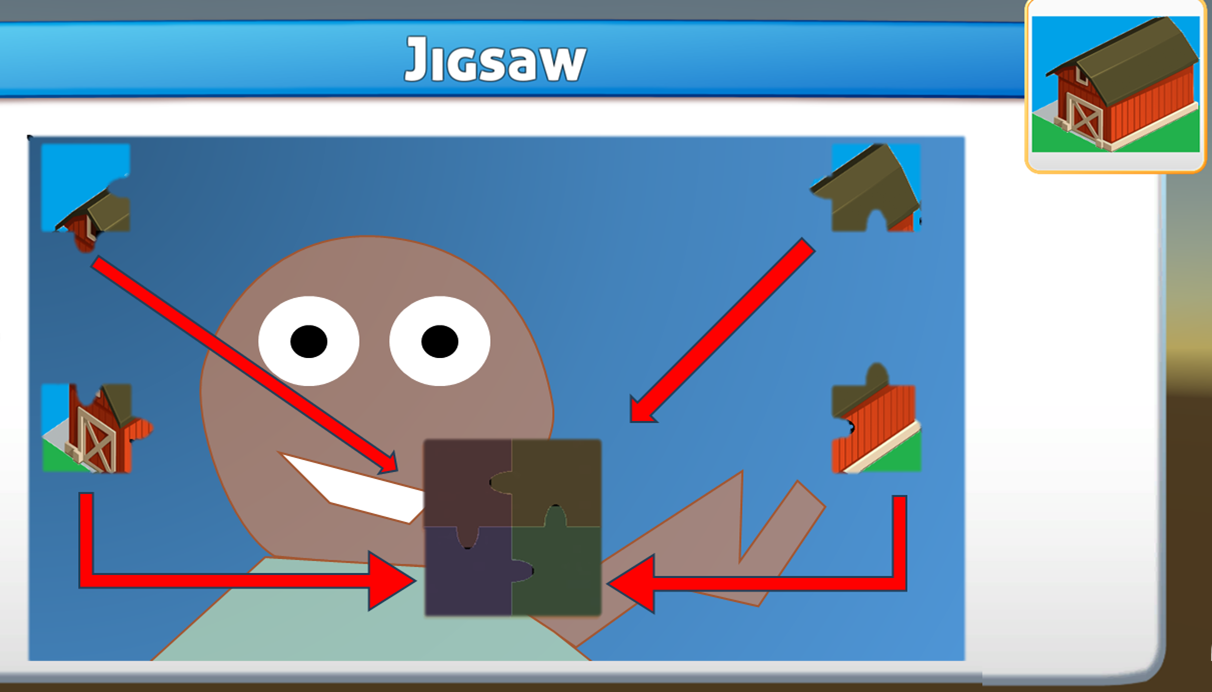** |

**3-** **Magic Pattern game:** this game aims to encourage the participant to improve their upper limb range of mobility. the participant will be instructed to draw a pattern in the form of following the guided hand and touching a ball at a specific point on the screen then touching the next target virtually until finishing the whole shape. This game contains more than difficulty levels starting with touching 2 balls to form a line and then increasing the number of balls and the complexity of the shape by changing the position of the balls that can reflect on the range of motion of the whole upper limb. The game also provides sensory feedback (visual – hit and miss feedback; auditory – anticipatory and delay error -proprioceptive stimulation in the form of stimulating the joint proprioception). the participant will be asked to move his hand trying to touch the ball when using hand movement (from sitting or standing positions). **Fig 5 (a,b)**.


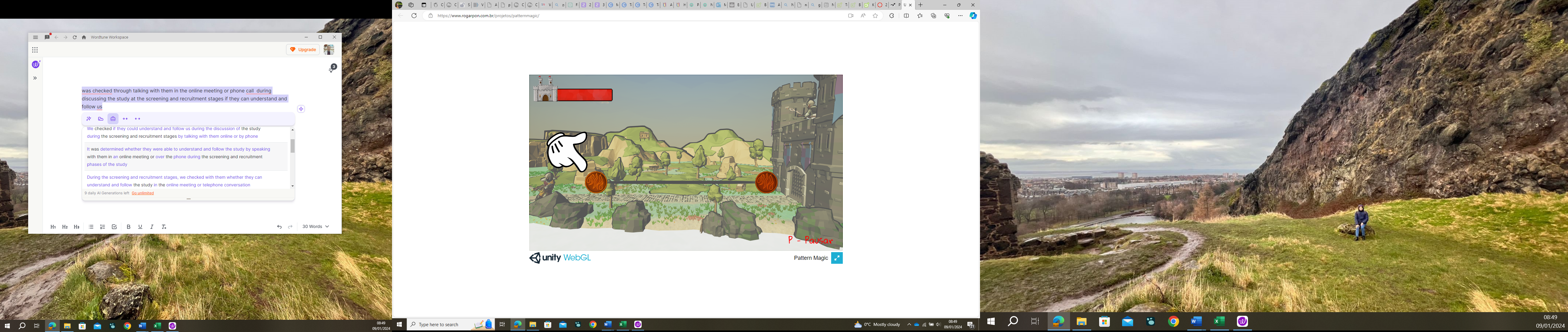


**Fig 5 (a): Magic Pattern game (Easy level)**


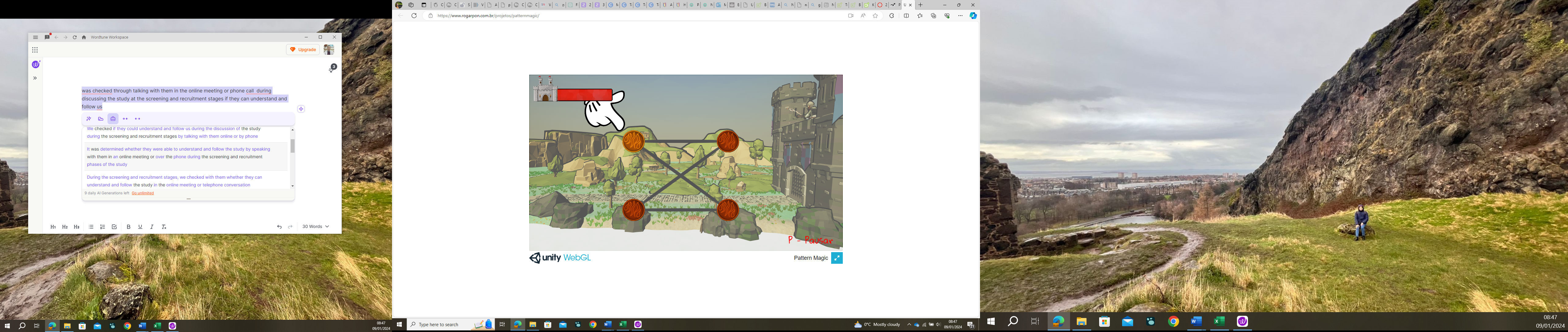


**Fig 5 (b): Magic Pattern game (Difficult level)**

| **Magic Pattern game** | |
| --- | --- |
| Step 1 | **Click on the Magic Pattern game link. https://rogarpon.com.br/projetos/patternmagic** |
| Step 2:   - In the first screen, click on JOGAR button to start the game. | 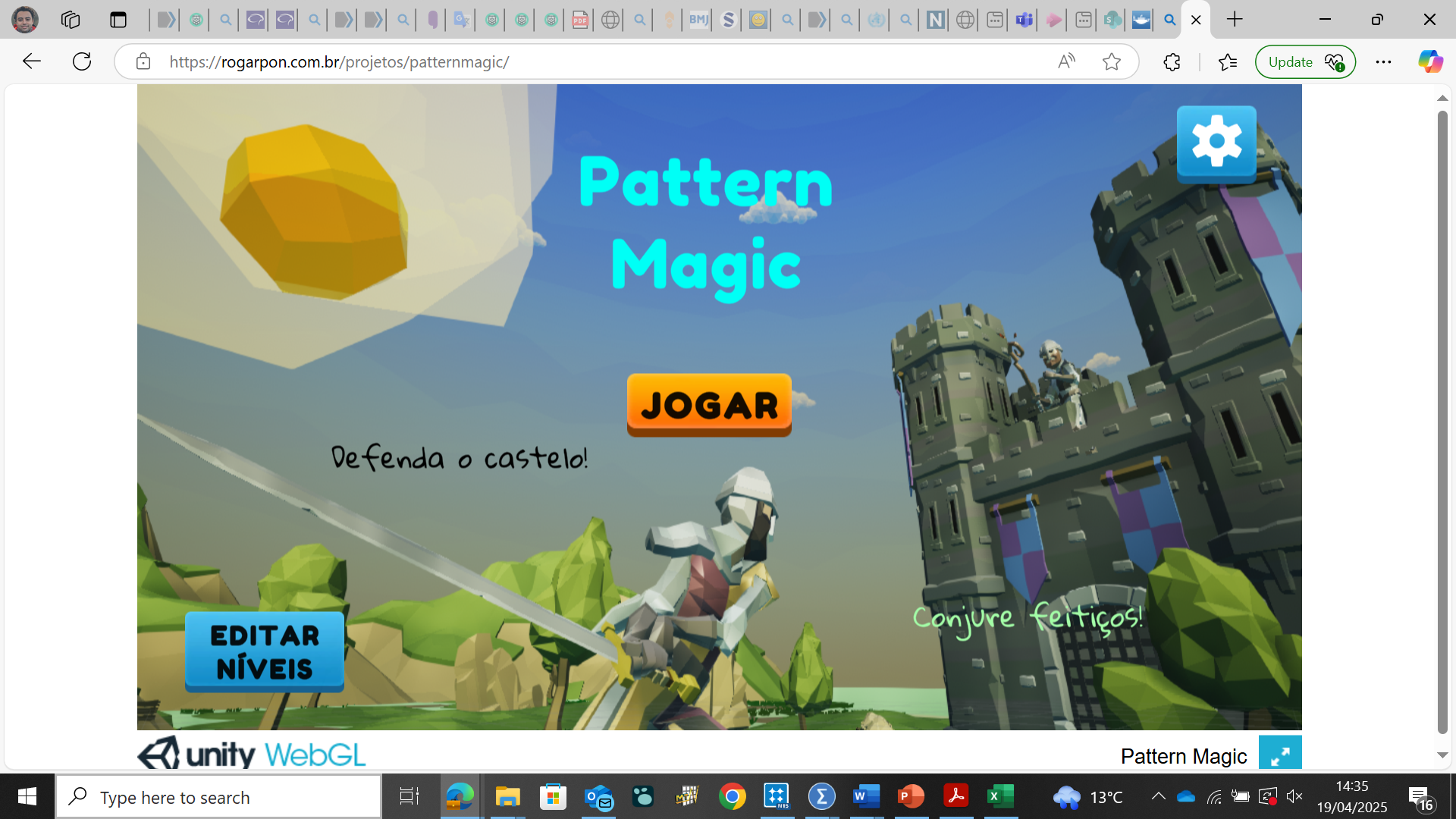  **Choose this** |
| - When you start the game, this page will appear on the screen. You will appear as a mirror avatar on the screen. Your head should be in the middle of the screen. You should be away from the screen with a distance about 1.5 meters. - **The task:** you will be instructed to draw a pattern in the form of following the guided hand and touching a ball at a specific point on the screen then touching the next target virtually until finishing the whole shape. This game contains more than difficulty levels starting with touching 2 balls to form a line and then increasing the number of balls and the complexity of the shape by changing the position of the balls that can reflect on the range of motion of the whole upper limb. You can do it from sitting or standing positions based on your physiotherapist recommendations. - **For the physiotherapists**, you will have to try doing these exercises from both sitting and standing position to be able to assess their usability as a telerehabilitation exercises for people with stroke. | 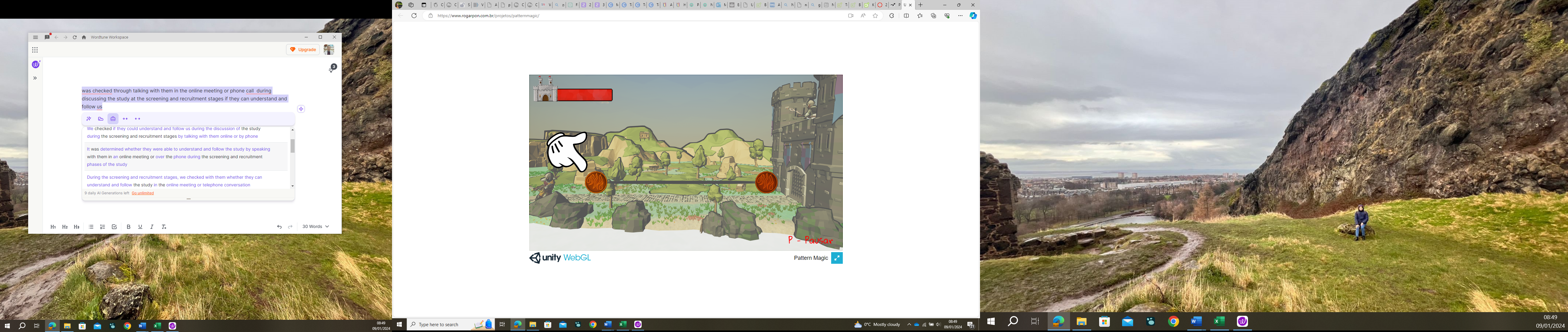 |

**4-Basketball game:** The participant is positioned in front of a computer and when the game starts the webcam captures the participant’s movements and a representation of the player appears on the computer screen as an avatar. The goal of the game is to try throwing the ball using upper limb throwing and reaching movements from either sitting or standing positions while the opponents’ virtual avatar lowers their hands. The game also provides sensory feedback (visual – hit and miss feedback; auditory – anticipatory and delay error) – if the individual reaches the basket correctly, On the other hand, if the participant does not reach the goal correctly, the letter X appears on the screen the participant will lose a point on the score together with a sound indicating an error. **Fig 6**


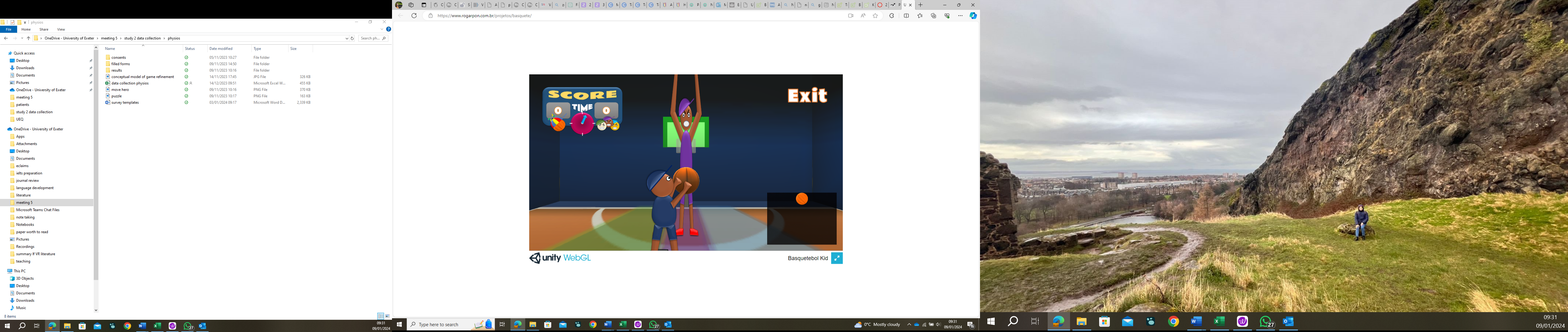


**Fig 6: Basketball game.**

| **Basketball game** | |
| --- | --- |
| **Step 1** | **Click on the Basketball game link: https://rogarpon.com.br/projetos/basquete/** |
| **Step 2:**   - Click on EN to choose the English language, then click on challenges. | **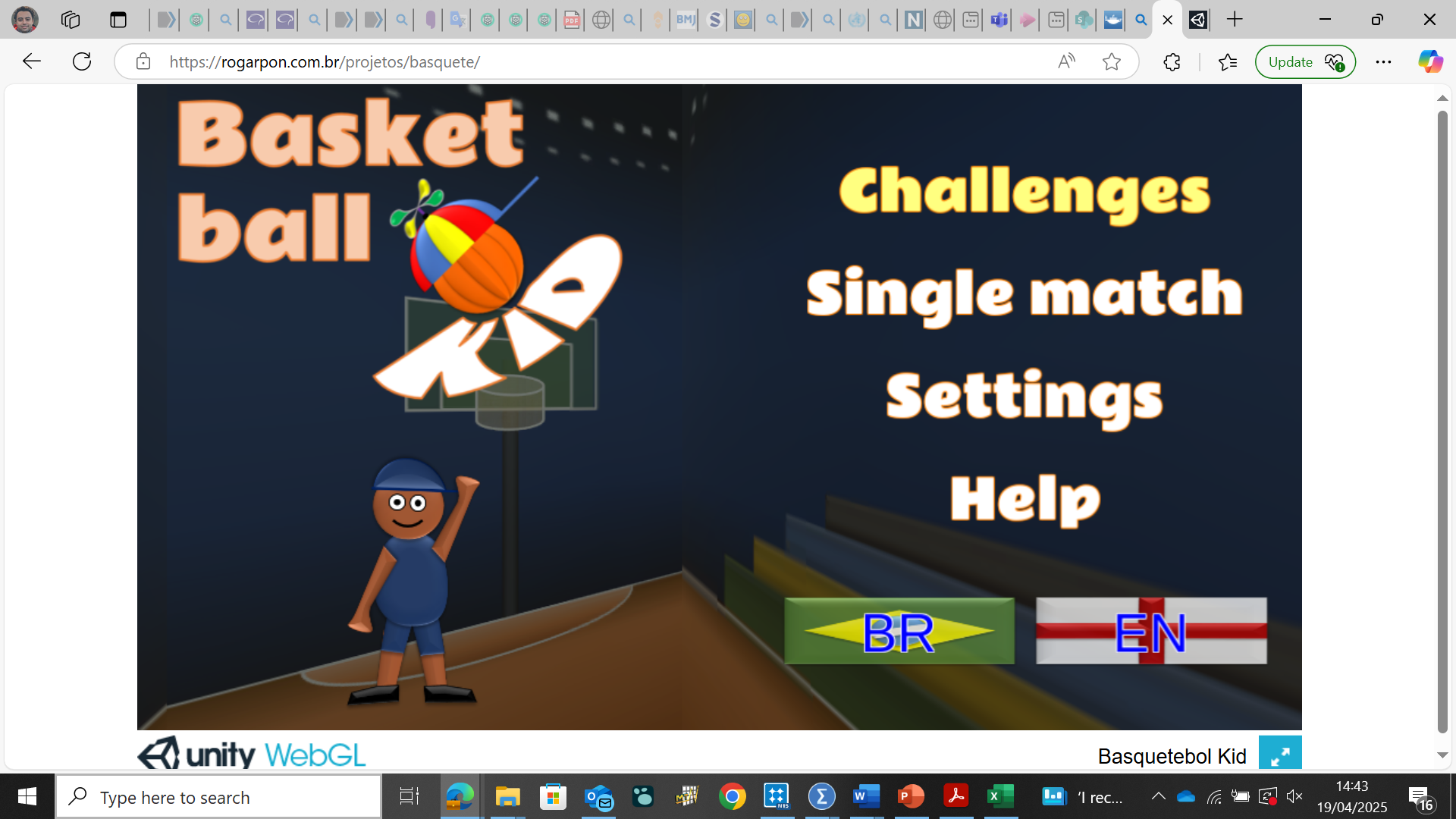**  **Click here** |
| **Step 3:**   - From challenges icon, you can choose from challenge 1 (easy levels, to challenge 4 (difficult levels). In each challenge, there are 10 different difficulty levels. - **For stroke survivors,** the researcher will decide which levels you can try. **For the physiotherapists,** they have to try different levels of difficulties to assess the suitability of this game to be a telerehabilitation tool for people with stroke. - In each challenge, there will be 10 levels. You have to start from level 1 then progress to the next level, until you finish all the 10 levels. | **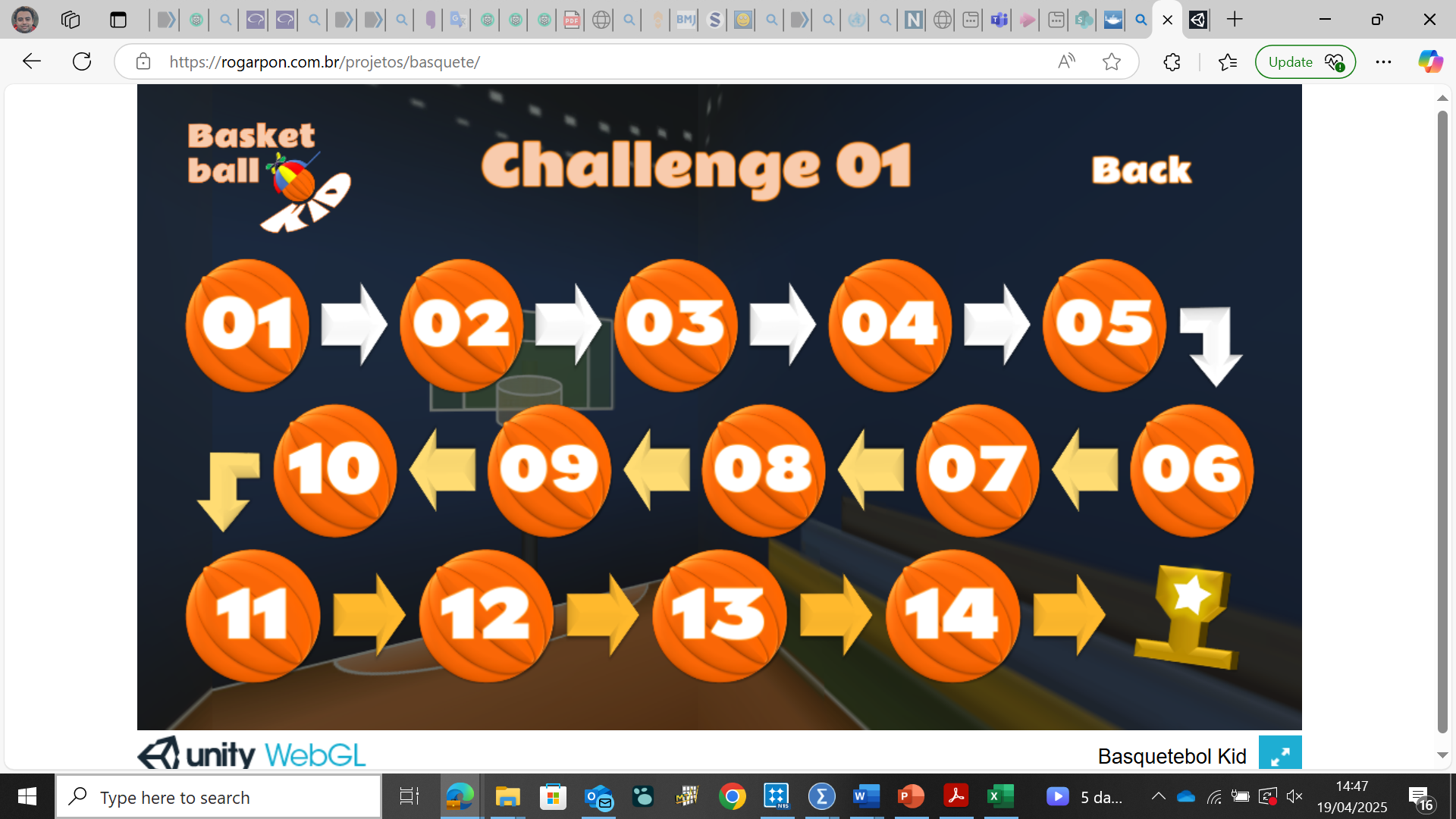** |
| **Step 4:**   - After choosing the level that you want to play, this screen will appear. - Choose Yes, I participate, so you can add your ID. - Add your ID inside the “Identifier” field. - Add the research password in the second field (REHABEXETER). - Click Start. | **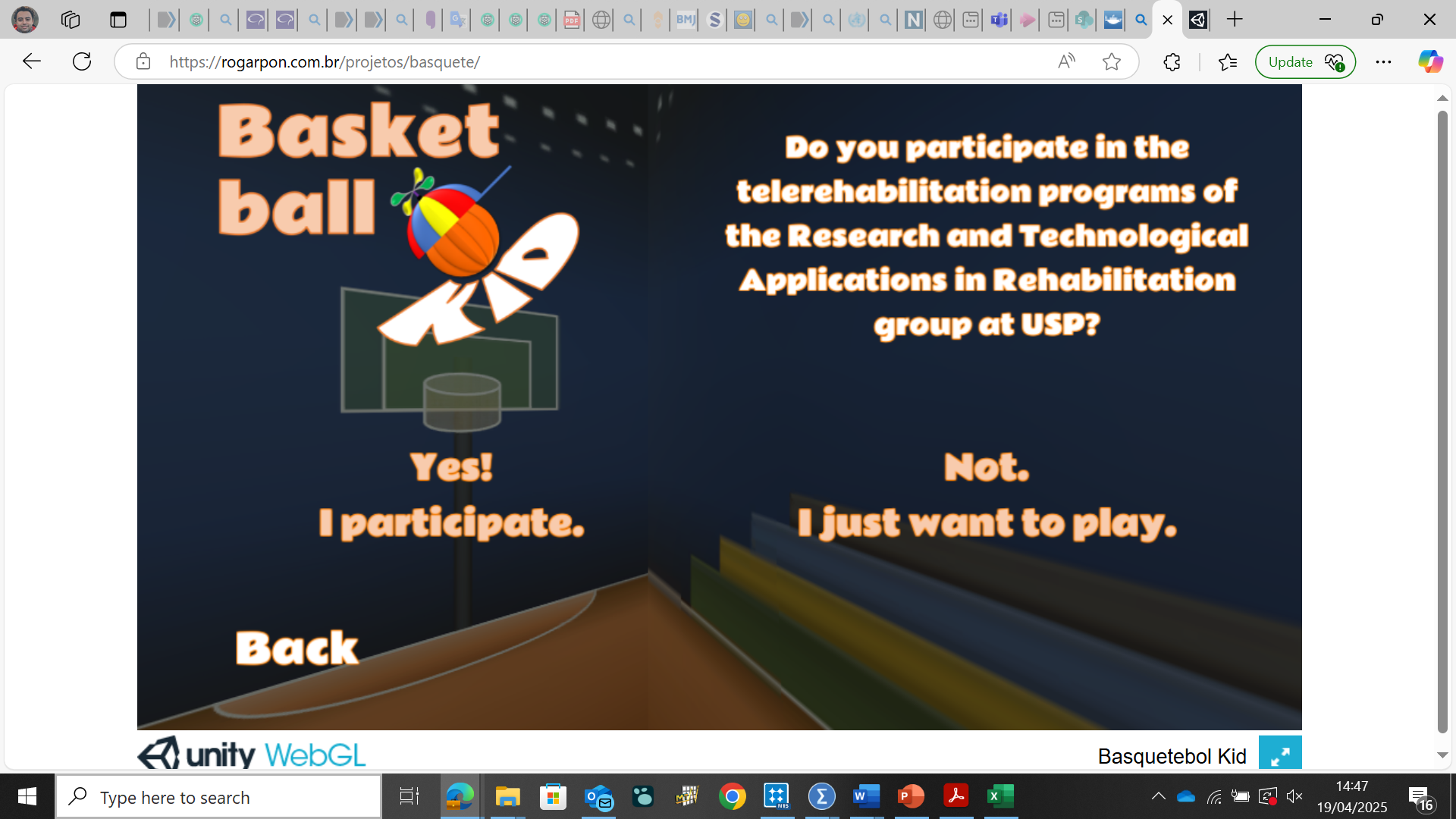**  **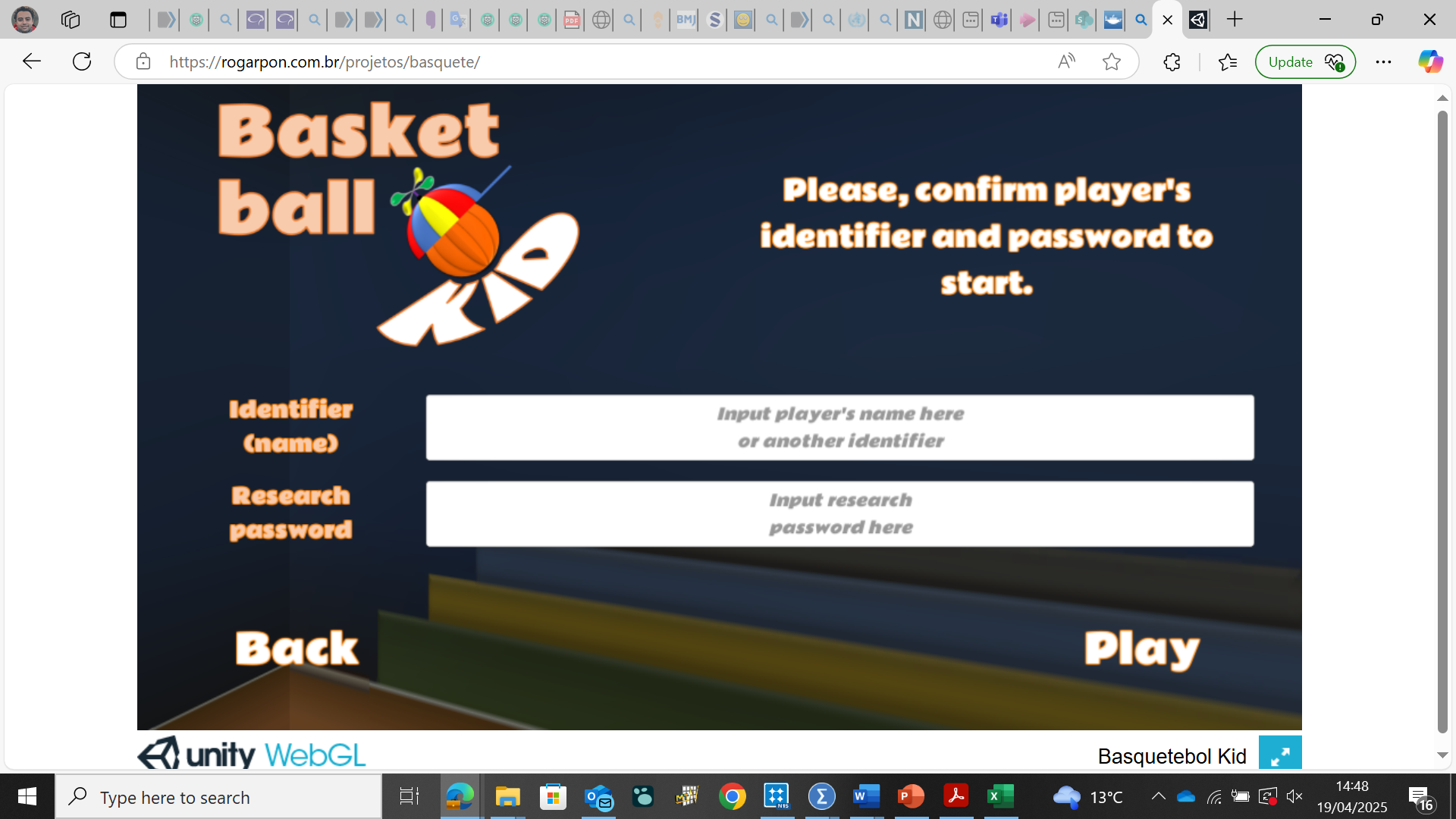** |
| - When you start the session, you can see yourself in the lower right side of the screen. You have to move your affected arm and reach the ball on the top of the small screen against the opponents’ avatar. - Your head should be in the middle of the small screen. You should be away from the screen with a distance about 1.5 meters. - You have to focus on the position of the opponent’s avatar arms to avoid any miss hitting. - By moving from level to another level, the position of the ball will change on the top part of the small screen. | **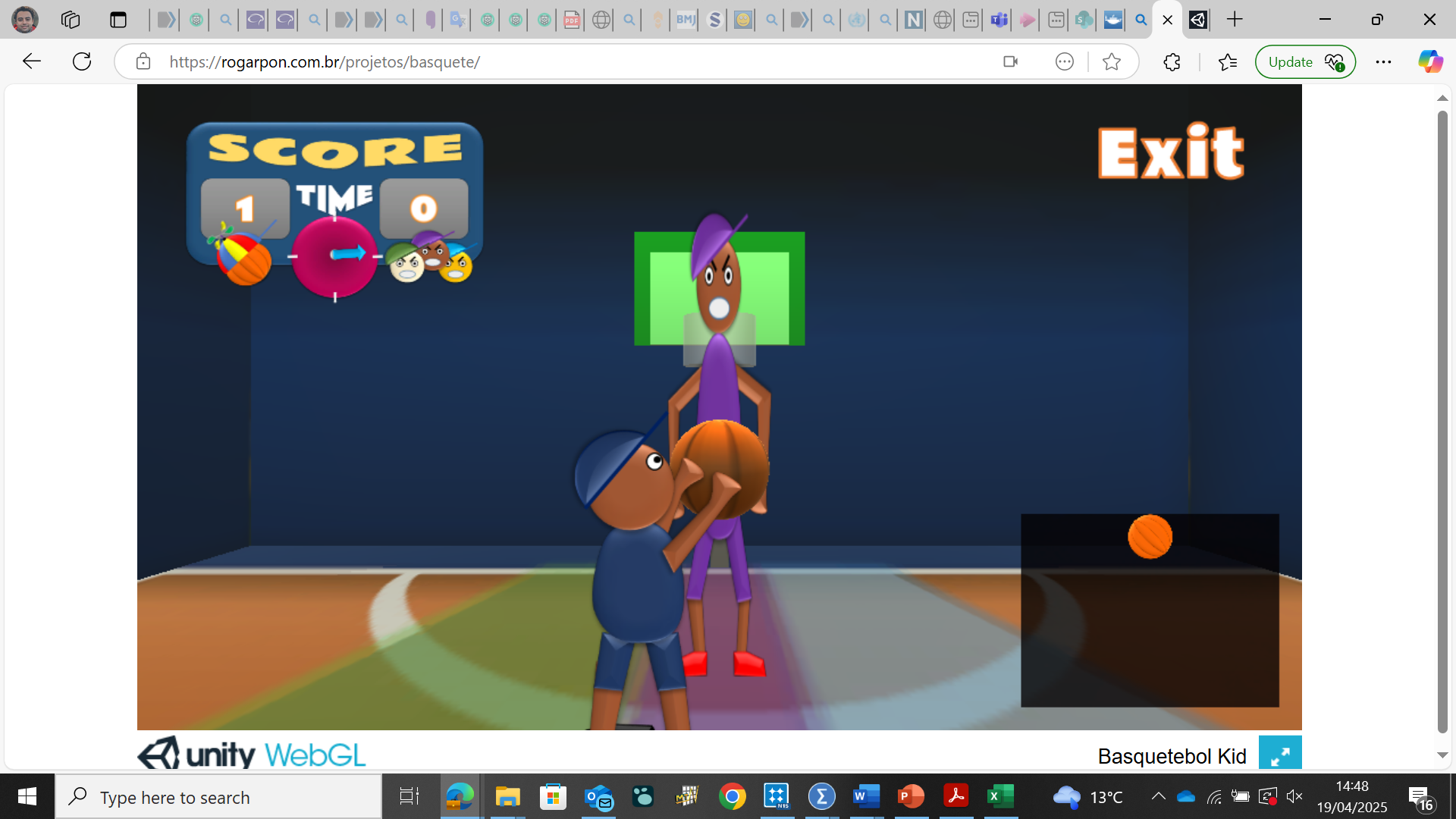**  Small screen |
| **Note** | For the flowers game, you will have to follow the same steps (similar to the basketball game, but different interactive environment). |

**5-Flowers game:** This game is the same as the basketball game, but the virtual environment is collecting flowers rather than throwing a ball. While this game has the same tasks as the previous games, the reason for adding it during the testing process was that in the PPI (patient and public involvement) meeting with stroke survivors and physiotherapists, it was recommended that different games with different motivating environments be included to give stroke patients several options for practicing their exercises depending on their motivation preference. **Fig 7**


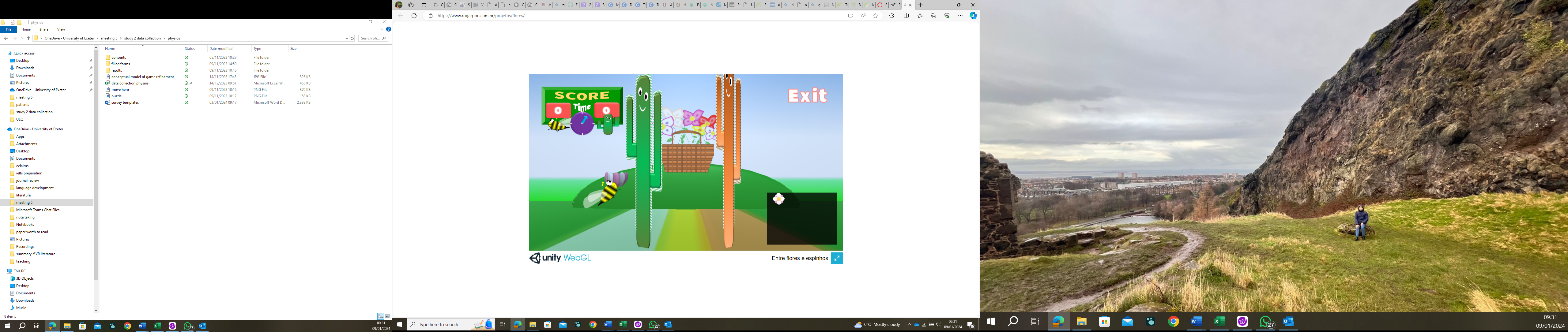


**Fig 7: Flowers game.**
