# Supplementary material for: Validity, safety, usability, and user experience of virtual reality gamified home-based exercises in stroke
Source: Clin Rehabil. 2025 Sep 2;39(11):1527–40. doi: 10.1177/02692155251371435 (PMC12521778; doi:10.1177/02692155251371435)
Supplement: sj-docx-2-cre-10.1177_02692155251371435 - Supplemental material for Validity, safety, usability, and user experience of virtual reality gamified home-based exercises in stroke [file sj-docx-2-cre-10.1177_02692155251371435.docx]

**Supplementary document 2**

***House of Quality Analysis***

The house of quality matrix consisted of:

1. User expectations survey: we asked the participant to fill out a survey before starting the testing session to rate their expectations among 10 criteria. This survey was designed by the research team and previously used in a similar usability study [1].
2. Satisfaction evaluation survey: After each game, a post-game patients’ satisfaction survey has been completed by the patients.
3. Technical characteristics: This part included the most important technical features in our intervention that may require technical improvement. These criteria have been selected by the development and research teams and guided by previous research [2].
4. Interrelationship matrix: in this part, the research team build a consensus to rate the strength of the relationship between the technical features and the user expectation criteria giving them a number that will help to calculate the priority weight for each of the technical features in each game [3].

***Pre-session assessment***

**Pre-game survey about patients’ expectations:**

*To meet your needs and fulfill your expectations, we're going to assess 10 items regarding how important they are to be included in remote rehabilitation exercises using technology.*

| 10 criteria | 1= not important, 2 = marginally important, 3= somewhat important, 4= important, and 5=very important |
| --- | --- |
| Easy to understand |  |
| Easy to use |  |
| Interesting |  |
| Motivating |  |
| Adequate difficulty |  |
| Graphics quality |  |
| Game variety |  |
| Score training (at the end of the game) |  |
| Clinical feedback (getting feedback based on a clinical assessment or clinical measures and tracking their clinical score) |  |
| Improve functional abilities and efficacy. |  |

**Post-game survey about patients’ satisfaction:**

*In this survey, we'd like to know if you're satisfied with the exercises in this game compared to your previous expectations.*

| ‘Game 1 (move hero)’ | |
| --- | --- |
| 10 criteria | 1 = terrible, 2 = unsatisfactory, 3 = okay, 4 = satisfactory, and 5 = exceeded expectation |
| Easy to understand |  |
| Easy to use |  |
| Interesting |  |
| Motivating |  |
| Adequate difficulty |  |
| Graphics quality |  |
| Game variety |  |
| Score training (at the end of the game) |  |
| Clinical feedback (getting feedback based on a clinical assessment or clinical measures and tracking their clinical score) |  |
| Improve functional abilities and efficacy. |  |
| If you have any comments about the games, what aspects would you like to share with us?  What aspects, if any, could be improved? | |

| Game 1  ‘Move Hero’ | **Priority Weight** | **45** | **45** | **29** | **60** | **56** | **36** | **29** | **15** | **53** | **23** |
| --- | --- | --- | --- | --- | --- | --- | --- | --- | --- | --- | --- |
|  | **Priority percent** | **12** | **12** | **7** | **15** | **14** | **9** | **7** | **4** | **14** | **6** |
|  | **Rank** | **3** | **3** | **5** | **1** | **2** | **4** | **5** | **7** | **2** | **6** |
| Game 2  ‘Puzzle’ | **Priority Weight** | **113** | **135** | **80** | **156** | **94** | **60** | **43** | **74** | **107** | **45** |
|  | **Priority percentage** | **12** | **15** | **9** | **17** | **10** | **7** | **5** | **8** | **12** | **5** |
|  | **Rank** | **3** | **2** | **5** | **1** | **4** | **7** | **8** | **6** | **3** | **8** |
| Game 3  ‘Magic Pattern’ | **Priority Weight** | **105** | **135** | **173** | **220** | **111** | **131** | **107** | **170** | **177** | **104** |
|  | **Priority percentage** | **7** | **8** | **11** | **14** | **7** | **8** | **7** | **11** | **11** | **7** |
|  | **Rank** | **4** | **3** | **2** | **1** | **4** | **3** | **4** | **2** | **2** | **4** |
| Game 4  ‘Basketball’ | **Priority Weight** | **60** | **135** | **89** | **98** | **56** | **51** | **44** | **78** | **98** | **68** |
|  | **Priority percentage** | **8** | **17** | **11** | **13** | **7** | **7** | **6** | **10** | **13** | **9** |
|  | **Rank** | **6** | **1** | **3** | **2** | **7** | **7** | **8** | **4** | **2** | **5** |
| Game 4  ‘Basketball’ | **Priority Weight** | **30** | **113** | **122** | **91** | **74** | **98** | **84** | **96** | **108** | **81** |
|  | **Priority percentage** | **3** | **13** | **14** | **10** | **8** | **11** | **9** | **11** | **12** | **9** |
|  | **Rank** | **8** | **2** | **1** | **5** | **7** | **4** | **6** | **4** | **3** | **6** |

| ***‘Ө’*** | ***Strong relationship (9)*** |
| --- | --- |
| ***‘O’*** | ***Moderate relationship (3)*** |
| ***‘△’*** | ***Weak relationship (1)*** |

| ***User***  ***Expectations*** | | ***Technical Characteristics*** | | | | | | | | | | ***Evaluation***  ***Rating*** | | | | |
| --- | --- | --- | --- | --- | --- | --- | --- | --- | --- | --- | --- | --- | --- | --- | --- | --- |
|  |  | ***Setting up instructions*** | ***Game instructions*** | ***Exercises scenarios*** | ***Avatar quality*** | ***Game reliability*** | ***Game adaptation*** | ***Levels of difficulties*** | ***Motivational Features*** | ***Clinical assessment*** | ***Clinical efficacy*** | ***Game 1 ‘Move Hero’*** | ***Game 2 ‘Puzzle’*** | ***Game 3 ‘Magic Pattern’*** | ***Game 4 ‘Basketball’*** | ***Game 5 ‘Flowers’*** |
| ***Criteria*** | ***Weight*** |  |  |  |  |  |  |  |  |  |  |  |  |  |  |  |
| Easy to understand | **5** | O | Ө | O | △ |  |  |  |  |  |  | 5 | 3.5 | 4 | 4 | 4.5 |
| Easy to use | **5** | Ө |  |  | Ө |  |  |  |  |  |  | 4 | 3 | 3 | 4 | 4.5 |
| Interesting | **5** |  | Ө | O | O | O | O | O | O |  |  | 4 | 4 | 3 | 4 | 4 |
| Motivating | **5** |  | Ө | Ө | O |  | O | O | Ө | Ө | Ө | 5 | 4.5 | 3.5 | 4 | 4 |
| Adequate difficulty | **4.5** |  |  | Ө |  |  | Ө | Ө |  | △ | O | 5 | 5 | 4 | 5 | 4 |
| Graphics quality | **4** |  |  |  | Ө |  |  |  | Ө |  |  | 5 | 4 | 3 | 4.5 | 4 |
| Game variety | **4.5** |  |  | O |  |  | △ | O |  |  |  | 4 | 3.5 | 4 | 4 | 4 |
| Score tracking | **4** |  |  |  |  | Ө | O |  |  | O |  | 4 | 3 | 3 | 4 | 3.5 |
| Clinical feedback | **4.5** |  |  |  |  | △ | △ |  |  | Ө |  | 4 | 3.5 | 3 | 4 | 4 |
| Improve functional abilities and efficacy. | **5** |  |  |  |  |  |  |  |  |  | Ө | 4.5 | 4.5 | 4.5 | 4.5 | 4.5 |

**Figure (1)** House of Quality matrix determined priority weights and percentages as outcomes based on user expectation weights for individual criteria, evaluation ratings for each game, technical characteristics, and interrelationship matrix. User expectation weights and evaluation ratings were median ratings obtained in pre- and postgame surveys, respectively.

***Content validity questionnaire***

*This survey will help us to understand the suitability of these exercises for stroke rehabilitation at home.*

| exercises | The introduction for the equipment and its function was clear (laptop or tablet camera, webcam, T.V screen.  (Setting up) | Instructions for the task before doing the exercises are clear. | Exercise is safe | Movement quality is good | motivational environment is good. | Exercises are suitable for balance training (sitting position) | Exercises are suitable for balance training (standing position) | Exercises are suitable for upper limb training. |
| --- | --- | --- | --- | --- | --- | --- | --- | --- |
| Game 1 (move hero)  https://movehero.com.br/en/ |  |  |  |  |  |  |  |  |
| Game 2 (Puzzle)  https://www.paterland.com/jigsaw/ |  |  |  |  |  |  |  |  |
| Game 3 (Pattern Magic)  https://rogarpon.com.br/projetos/patternmagic |  |  |  |  |  |  |  |  |
| Game 4 (Basketball)  https://rogarpon.com.br/projetos/basquete/ |  |  |  |  |  |  |  |  |
| Game 5 (Flowers)  https://rogarpon.com.br/projetos/flores/ |  |  |  |  |  |  |  |  |

| Open-ended questions |
| --- |
| What do you think about exercise progression and difficulty levels? |
| What do you think about the suitability of these exercises to be part of the balance, upper limb training for stroke rehabilitation at home? |
| What aspects, if any, could be improved? |

To calculate the content validity index(CVI) for each item agreement (I-CVI), the number of experts giving a rating of either 3 or 4 was divided by the total number of experts [4]. To calculate the CVI for each game among all criteria (AVE-CVI), the sum of item-CVI (I-CVI ) scores was divided by the number of items [4]. It is recommended that CVIs should be ≥ 0.78 to have a good agreement among the expert opinions [5]. For the qualitative data obtained from the open-ended questions, content analysis was used to determine the main areas for refinement [6].

Before calculating the CVI, the relevant rating was recoded as 1 (for 3 or 4 rating) or 0 (for 1 or 2 rating).

| **Move Hero** | **Introduction for the equipment and its function was clear** | **Instructions for the task are clear** | **Exercise is safe** | **Movement quality is good** | **motivational environment is good.** | **Exercises are suitable for balance training (sitting position)** | **Exercises are suitable for balance training (standing position)** | **Exercises are suitable for upper limb training.** |
| --- | --- | --- | --- | --- | --- | --- | --- | --- |
| P1 | 0 | 0 | 1 | 0 | 0 | 1 | 1 | 1 |
| P2 | 1 | 1 | 1 | 0 | 1 | 1 | 1 | 1 |
| P3 | 0 | 0 | 1 | 1 | 0 | 0 | 0 | 1 |
| P4 | 1 | 1 | 1 | 0 | 1 | 0 | 1 | 1 |
| P5 | 1 | 0 | 1 | 1 | 1 | 1 | 1 | 1 |
| P6 | 1 | 1 | 1 | 0 | 1 | 0 | 1 | 0 |
| P7 | 1 | 1 | 1 | 1 | 1 | 0 | 1 | 1 |
| P8 | 0 | 0 | 1 | 1 | 0 | 1 | 1 | 0 |
| P9 | 0 | 0 | 1 | 0 | 1 | 1 | 1 | 1 |
| P10 | 1 | 1 | 1 | 1 | 1 | 1 | 1 | 1 |
| P11 | 1 | 1 | 1 | 1 | 1 | 1 | 1 | 1 |
|  |  |  |  |  |  |  |  |  |
| experts in agreement | 7 | 6 | 11 | 6 | 8 | 7 | 10 | 9 |
| **I-CVI** | **0.63** | **0.54** | **1** | **0.54** | **0.72** | **0.63** | **0.9** | **0.81** |
| **S-CVI/Ave** | **0.72125** |  |  |  |  |  |  |  |

| **Puzzle** | **Introduction for the equipment and its function was clear** | **Instructions for the task are clear** | **Exercise is safe** | **Movement quality is good** | **motivational environment is good.** | **Exercises are suitable for balance training (sitting position)** | **Exercises are suitable for balance training (standing position)** | **Exercises are suitable for upper limb training.** |
| --- | --- | --- | --- | --- | --- | --- | --- | --- |
| P1 | 0 | 0 | 1 | 0 | 0 | 1 | 1 | 1 |
| P2 | 1 | 1 | 1 | 0 | 1 | 1 | 1 | 1 |
| P3 | 0 | 0 | 1 | 1 | 0 | 0 | 0 | 1 |
| P4 | 1 | 1 | 1 | 1 | 1 | 1 | 1 | 1 |
| P5 | 1 | 1 | 1 | 1 | 1 | 0 | 0 | 1 |
| P6 | 1 | 1 | 1 | 1 | 1 | 1 | 1 | 1 |
| P7 | 0 | 0 | 1 | 0 | 0 | 0 | 1 | 1 |
| P8 | 1 | 1 | 1 | 1 | 1 | 1 | 1 | 1 |
| P9 | 1 | 1 | 1 | 1 | 1 | 1 | 1 | 1 |
| P10 | 1 | 1 | 1 | 1 | 1 | 1 | 1 | 1 |
| P11 | 1 | 1 | 1 | 1 | 1 | 1 | 1 | 1 |
|  |  |  |  |  |  |  |  |  |
| experts in agreement | 8 | 8 | 11 | 8 | 8 | 8 | 9 | 11 |
| I-CVI | **0.72** | **0.72** | 1 | **0.72** | **0.72** | **0.72** | **0.81** | **1** |
| **S-CVI/Ave** | **0.80125** |  |  |  |  |  |  |  |

| **Magic pattern** | **Introduction for the equipment and its function was clear** | **Instructions for the task are clear** | **Exercise is safe** | **Movement quality is good** | **motivational environment is good.** | **Exercises are suitable for balance training (sitting position)** | **Exercises are suitable for balance training (standing position)** | **Exercises are suitable for upper limb training.** |
| --- | --- | --- | --- | --- | --- | --- | --- | --- |
| P1 | 0 | 0 | 1 | 0 | 0 | 0 | 0 | 0 |
| P2 | 1 | 0 | 1 | 1 | 1 | 1 | 1 | 1 |
| P3 | 1 | 0 | 1 | 1 | 0 | 0 | 0 | 1 |
| P4 | 1 | 0 | 1 | 1 | 1 | 1 | 1 | 1 |
| P5 | 1 | 0 | 1 | 1 | 1 | 1 | 1 | 1 |
| P6 | 1 | 1 | 1 | 1 | 1 | 0 | 1 | 1 |
| P7 | 1 | 1 | 1 | 1 | 1 | 1 | 1 | 1 |
| P8 | 0 | 0 | 0 | 0 | 0 | 0 | 0 | 0 |
| P9 | 0 | 0 | 0 | 0 | 0 | 0 | 0 | 0 |
| P10 | 1 | 1 | 1 | 1 | 0 | 1 | 1 | 1 |
| P11 | 1 | 1 | 1 | 1 | 1 | 1 | 1 | 1 |
|  |  |  |  |  |  |  |  |  |
| experts in agreement | 8 | 4 | 9 | 8 | 6 | 6 | 6 | 8 |
| I-CVI | **0.72** | **0.36** | 0.81 | **0.72** | **0.54** | **0.54** | **0.54** | **0.72** |
| **S-CVI/Ave** | **0.61875** |  |  |  |  |  |  |  |

| **Basketball** | **Introduction for the equipment and its function was clear** | **Instructions for the task are clear** | **Exercise is safe** | **Movement quality is good** | **motivational environment is good.** | **Exercises are suitable for balance training (sitting position)** | **Exercises are suitable for balance training (standing position)** | **Exercises are suitable for upper limb training.** |
| --- | --- | --- | --- | --- | --- | --- | --- | --- |
| P1 | 0 | 0 | 1 | 0 | 0 | 1 | 1 | 1 |
| P2 | 1 | 1 | 1 | 1 | 0 | 1 | 1 | 1 |
| P3 | 1 | 0 | 1 | 1 | 1 | 1 | 1 | 1 |
| P4 | 1 | 1 | 1 | 1 | 1 | 0 | 1 | 1 |
| P5 | 1 | 1 | 1 | 1 | 1 | 1 | 1 | 1 |
| P6 | 1 | 1 | 1 | 1 | 1 | 1 | 1 | 1 |
| P7 | 1 | 1 | 1 | 1 | 1 | 1 | 1 | 1 |
| P8 | 1 | 1 | 1 | 1 | 1 | 1 | 1 | 1 |
| P9 | 1 | 1 | 1 | 1 | 1 | 1 | 1 | 1 |
| P10 | 1 | 1 | 1 | 1 | 1 | 1 | 1 | 1 |
| P11 | 1 | 1 | 1 | 1 | 1 | 1 | 1 | 1 |
|  |  |  |  |  |  |  |  |  |
| experts in agreement | 10 | 9 | 11 | 10 | 9 | 10 | 11 | 11 |
| I-CVI | **0.9** | **0.81** | **1** | **0.9** | **0.81** | **0.9** | **1** | **1** |
| **S-CVI/Ave** | **0.915** |  |  |  |  |  |  |  |

| **participant ID** | **Introduction for the equipment and its function was clear** | **Instructions for the task are clear** | **Exercise is safe** | **Movement quality is good** | **motivational environment is good.** | **Exercises are suitable for balance training (sitting position)** | **Exercises are suitable for balance training (standing position)** | **Exercises are suitable for upper limb training.** |
| --- | --- | --- | --- | --- | --- | --- | --- | --- |
| P1 | 0 | 0 | 1 | 0 | 0 | 1 | 1 | 1 |
| P2 | 1 | 1 | 1 | 1 | 0 | 1 | 1 | 1 |
| P3 | 1 | 0 | 1 | 1 | 0 | 1 | 0 | 1 |
| P4 | 1 | 1 | 1 | 1 | 1 | 0 | 1 | 1 |
| P5 | 1 | 1 | 1 | 1 | 1 | 1 | 1 | 1 |
| P6 | 1 | 1 | 1 | 1 | 0 | 1 | 1 | 1 |
| P7 | 1 | 0 | 1 | 1 | 1 | 1 | 1 | 1 |
| P8 | 1 | 1 | 1 | 1 | 1 | 1 | 1 | 1 |
| P9 | 1 | 1 | 1 | 1 | 1 | 1 | 1 | 1 |
| P10 | 1 | 1 | 1 | 1 | 1 | 1 | 1 | 1 |
| P11 | 1 | 1 | 1 | 1 | 1 | 1 | 1 | 1 |
|  |  |  |  |  |  |  |  |  |
| experts in agreement | 10 | 8 | 11 | 10 | 7 | 10 | 10 | 11 |
| I-CVI | **0.9** | **0.72** | **1** | **0.9** | **0.63** | **0.9** | **0.9** | **1** |
| **S-CVI/Ave** | **0.86875** |  |  |  |  |  |  |  |

**Qualitative analysis**

Content analysis was used to analyse the experts’ answers regarding the exercise progression, difficulty levels, suitability for telerehabilitation, and any suggestions for future improvement. Based on the responses from the experts, we can categorize their feedback into six main categories: instructions of the exercises, accuracy of the games, exercise safety, movement quality, motivation, and exercise scenarios.

1-Instructions of the exercises: all the included games didn’t include a clear instruction for how to start the games and set up the needed equipment, and lack for the instructions for the physiotherapists on how to tailor the exercises program according to the patient abilities ‘*More instructions to be added, and options for difficulty adjustments*’, and the instructions to the patients on hot to perform the exercises accurately ‘*Clearer instructions on how to perform the game*’.

2-Accuracy of the game: two games (Move Hero and Pattern Magic) showed a less clear avatar that can easily be affected by the lighting of the room and the position of the camera. This can in turn have a negative effect on task execution during playing the game and induce a lot of false errors *’Move-Hero game: Actually, the webcam doesn’t work well. My forearm often touches the target and lose the score’, ‘Magic Pattern game: The game did not response to my movements’*. This can affect the internal motivation of the patient and their desire to practice the exercises for a higher number or repetitions. The other three games showed a good quality regarding the accuracy of the graphics as the camera can detect the whole pictures of the body not just an avatar. Most of the experts recommend that it will be useful if these games can detect the isolated hand movement so the score should only be correct if the participant hit the target with their hands rather that hitting the target with any other body parts.

3-Exercise safety: all experts recommended that all exercises included in these five games can be safe to be administrated as part of the upper limb and balance training as a telerehabilitation tool as the patients can do these exercises from sitting or standing position with many levels of difficulties according to their abilities so the patient has low liability to do a substitution which can influence their safety at home, however there should be a clear instruction about how to do the exercises to avoid any risks such as falling *‘basketball game: I would make sure the patient is safe from falling’.*

4*-*Movement quality: to achieve a good quality movement, the patient should do the exercises tailored according to their abilities to avoid doing lot of substitutions and movement cheating. Most of the included games need some adaptation for some parameters *‘Move Hero:* *Easy and medium levels are proper for stroke patients, but hard and expert levels are too fast’ ‘Puzzle: the easiest levels are very frustrating trying to get the puzzle piece to move where you wanted it to. I think it would get very frustrating very quickly, the more complex it became the more frustrating it was trying to get them in the right places”.* They suggested that if the patient gets frustrated from doing the exercises due to the inappropriate parameters that could lead to movement substitutions and bad movement quality.

5-Performance feedback: despite that all the games give sensory feedback in form of auditory and visual feedback; it can only give feedback regarding the movement performance . There is lack in the tactile stimulation which is one of the important sensory types of feedback during the recovery process ‘Move Hero: *There is no tactile sensory reward for achieving the target*’. All of the included games didn’t provide any feedback regarding the kinematic performance of the upper limb that can affect the patient’s motivation to practice the exercises alone.

6-Exercises scenarios: this theme can be sub-classified into 3 categories; difficulty levels, progression of exercises, aims of exercises.

- Difficulty levels: there was a debate regarding the difficulty of the levels in some games (Move Hero, Magic Pattern, Basketball) and their suitability for stroke people, however, all games showed an acceptable level of difficulties despite some of them requiring some adjustment to be more tailored according to stroke patients’ abilities ‘*Move Hero: Easy and medium levels are proper for stroke patients, but hard and expert levels are too fast- Easy level quite hard’, ‘Magic pattern: The difficulty level can improve speed, response, and memory for remembering movement order, including upper limb coordination’, ‘Basketball: The adversaries velocity is still too fast for stroke patients even if the setting is the lowest - The difficulty level can improve the response time and cognitive function as well as decision making.’*.
- Progression of exercises: all the games except the puzzle game progress the levels of challenges based on the score of the patient and the success rate after each trial.
  - - For the Move Hero game, the progression levels was good but not too challenging for the patient and mainly focused only on improving the response and decision making with no progression regarding the kinematics of the upper limb *‘I quickly became bored with it - Improving response and decision-making can be facilitated by the level of progression.’*
    - For the Puzzle game, there was an agreement that there were no instructions on how to move to the more difficult levels and the parameters of difficulty was not clear ‘*I could not find any difficulty levels - The explanation of adjusting difficulty is unclear, I didn’t even know the difficulty is changeable at first*.’.
    - Magic Pattern game reported to have a good progression level ‘*There are a lot of levels to move through so seems there is a lot of scope for progression with this one’*, however they suggested to add more improvement ‘*The difficulty level can improve speed, response, and memory for remembering movement order, including upper limb coordination.*’.
    - Basketball and flowers games showed a good progression system regarding the speed and the coordination, but some experts suggested that we can modify the progression to be slower to be more suitable for stroke people that usually have problems in their coordination and speed of movement ‘*The game basketball and flowers did the best in difficulty setting, with suitable progression in each grade.*’.
- Aim of exercises: most of the exercises focused on the upper limb training and balance from sitting and standing positions. Some of the expert reported that these games can be suitable for upper limb training but not for balance training *‘Move Hero:* *Seems to me suitable for upper limb and balance while patient standing more sitting. – Magec Pattern:* *training moves centre of mass too little’ or* can be suitable for both upper limb and balance rehabilitation. It was also reported that these games could improve the velocity, coordination of the upper limb movements and cognitive stimulation *‘Basketball:* *I think this is a good game for all round rehab – U/L, balance and also some cognitive stimulation’.*

All factors included in this theme could act as a barrier against doing these exercises or act as a facilitator that can encourage the patient to do their exercises with high repetitions at home.

**System usability scale**

Item analysis of the system usability scale(SUS). This figure shows the physiotherapist's and stroke patients' rating of the 10 criteria included in the system usability scale. The blue line shows the median score among the physiotherapists, the orange line shows the median score among the stroke patients, and the green line shows the best usability result.

**Item analysis of SUS scale. This figure shows the physiotherapist's and stroke patients' rating of the 10 criteria included in the system usability scale. The blue line shows the median score among the physiotherapists, the orange line shows the median score among the stroke patients, and the green line shows the best usability result.**

| (1) Move Hero Game | | | | |  |
| --- | --- | --- | --- | --- | --- |
| Scale | **Mean** | **Std. Dev.** | **Confidence interval** | | **Level of agreement** |
| Attractiveness | **⇧**1.93 | 0.89 | 1.43 | 2.43 | Moderate |
| Perspicuity | **⇧**2.23 | 0.70 | 1.83 | 2.63 | High |
| Efficiency | **⇧**2.10 | 0.83 | 1.64 | 2.57 | Moderate |
| Dependability | **⇧**1.10 | 0.72 | 0.70 | 1.51 | High |
| Stimulation | **⇧**1.65 | 0.73 | 1.23 | 2.06 | High |
| Novelty | **⇧**1.52 | 0.88 | 1.02 | 2.02 | Moderate |

**User experience questionaire(UEQ) analysis:**

| (1) Move Hero Game | | | | |  |
| --- | --- | --- | --- | --- | --- |
| Scale | **Mean** | **Std. Dev.** | **Confidence interval** | | **Level of agreement** |
| Attractiveness | **⇧**1.93 | 0.89 | 1.43 | 2.43 | Moderate |
| Perspicuity | **⇧**2.23 | 0.70 | 1.83 | 2.63 | High |
| Efficiency | **⇧**2.10 | 0.83 | 1.64 | 2.57 | Moderate |
| Dependability | **⇧**1.10 | 0.72 | 0.70 | 1.51 | High |
| Stimulation | **⇧**1.65 | 0.73 | 1.23 | 2.06 | High |
| Novelty | **⇧**1.52 | 0.88 | 1.02 | 2.02 | Moderate |

| (2) Puzzle Game | | | | | |
| --- | --- | --- | --- | --- | --- |
| Scale | **Mean** | **Std. Dev.** | **Confidence interval** | | **Level of agreement** |
| Attractiveness | **⇧**1.28 | 1.41 | 0.48 | 2.08 | Low |
| Perspicuity | **⇧**1.52 | 1.21 | 0.84 | 2.20 | Low |
| Efficiency | **⇧**1.10 | 1.14 | 0.46 | 1.75 | Low |
| Dependability | **⇧**1.19 | 1.03 | 0.60 | 1.77 | Low |
| Stimulation | **⇧**1.29 | 1.24 | 0.59 | 1.99 | Low |
| Novelty | **⇧**0.94 | 1.45 | 0.12 | 1.76 | Low |

| (3) Magic Pattern Game | | | | | | |
| --- | --- | --- | --- | --- | --- | --- |
| Scale | **Mean** | **Std. Dev.** | **Confidence interval** | | **Level of agreement** | |
| Attractiveness | **⇨**0.46 | 1.62 | -0.46 | 1.38 | | Low |
| Perspicuity | **⇨**0.71 | 1.77 | -0.29 | 1.71 | | Low |
| Efficiency | **⇨**0.79 | 1.36 | 0.02 | 1.56 | | Low |
| Dependability | **⇨**0.69 | 1.02 | 0.11 | 1.27 | | Low |
| Stimulation | **⇧**0.88 | 1.36 | 0.11 | 1.64 | | Low |
| Novelty | **⇧**1.04 | 1.03 | 0.46 | 1.62 | | Low |

| (4) Basketball Game | |  |  | |  |
| --- | --- | --- | --- | --- | --- |
| Scale | **Mean** | **Std. Dev.** | **Confidence interval** | | **Level of agreement** |
| Attractiveness | **⇧**1.75 | 1.06 | 1.15 | 2.35 | Low |
| Perspicuity | **⇧**1.67 | 1.05 | 1.07 | 2.26 | Low |
| Efficiency | **⇧**1.63 | 1.09 | 1.01 | 2.24 | Low |
| Dependability | **⇧**1.17 | 0.98 | 0.61 | 1.72 | Moderate |
| Stimulation | **⇧**1.96 | 0.82 | 1.50 | 2.42 | Moderate |
| Novelty | **⇧**1.73 | 0.69 | 1.34 | 2.12 | High |

| (5) Flowers Game | |  |  | |  |
| --- | --- | --- | --- | --- | --- |
| Scale | **Mean** | **Std. Dev.** | **Confidence interval** | | **Level of agreement** |
| Attractiveness | **⇧**1.75 | 0.71 | 1.35 | 2.15 | High |
| Perspicuity | **⇧**2.15 | 0.66 | 1.77 | 2.52 | High |
| Efficiency | **⇧**1.79 | 0.70 | 1.40 | 2.19 | High |
| Dependability | **⇧**1.19 | 0.97 | 0.64 | 1.73 | Moderate |
| Stimulation | **⇧**1.54 | 1.06 | 0.94 | 2.14 | Low |
| Novelty | **⇧**1.25 | 1.11 | 0.62 | 1.88 | Low |

**Table 1:** UEQ finding per scale (mean, standard deviation, confidence interval, level of agreement). ⇧ represent positive evaluation, and ⇨ represent neutral evaluation.

**UEQ Benchmark dataset:**

| **Scale** | **Lower Border** | **Bad** | **Below Average** | **Above Average** | **Good** | **Excellent** |
| --- | --- | --- | --- | --- | --- | --- |
| **Attractiveness** | -1.00 | 0.69 | 0.49 | 0.4 | 0.26 | 0.66 |
| **Perspicuity** | -1.00 | 0.72 | 0.48 | 0.53 | 0.27 | 0.5 |
| **Efficiency** | -1.00 | 0.6 | 0.45 | 0.45 | 0.38 | 0.62 |
| **Dependability** | -1.00 | 0.78 | 0.36 | 0.34 | 0.22 | 0.8 |
| **Stimulation** | -1.00 | 0.5 | 0.5 | 0.35 | 0.35 | 0.8 |
| **Novelty** | -1.00 | 0.16 | 0.54 | 0.42 | 0.48 | 0.9 |

| **Benchmark borders** | | | | |
| --- | --- | --- | --- | --- |
| **Scale** | **25%** | **50%** | **75%** | **90%** |
| **Attractiveness** | 0.69 | 1.18 | 1.58 | 1.84 |
| **Perspicuity** | 0.72 | 1.2 | 1.73 | 2 |
| **Efficiency** | 0.6 | 1.05 | 1.5 | 1.88 |
| **Dependability** | 0.78 | 1.14 | 1.48 | 1.7 |
| **Stimulation** | 0.5 | 1 | 1.35 | 1.7 |
| **Novelty** | 0.16 | 0.7 | 1.12 | 1.6 |

| Excellent | In the range of the 10% best results |
| --- | --- |
| Good | 10% of results better, 75% of results worse |
| Above average | 25% of results better, 50% of results worse |
| Below average | 50% of results better, 25% of results worse |
| Bad | In the range of the 25% worst results |

**References:**

1. Na Jin, S., et al., *Usability evaluation of low-cost virtual reality hand and arm rehabilitation games.* Journal of Rehabilitation Research & Development, 2016. **53**(3): p. 321-333.

2. Seo, N.J., et al., *Usability evaluation of low-cost virtual reality hand and arm rehabilitation games.* J Rehabil Res Dev, 2016. **53**(3): p. 321-34.

3. Park, T. and K.-J. Kim, *Determination of an optimal set of design requirements using house of quality.* Journal of operations management, 1998. **16**(5): p. 569-581.

4. Yusoff, M.S.B., *ABC of Content Validation and Content Validity Index Calculation.* Education in Medicine Journal, 2019. **11**: p. 49-54.

5. Polit, D.F. and C.T. Beck, *The content validity index: are you sure you know what's being reported? Critique and recommendations.* Res Nurs Health, 2006. **29**(5): p. 489-97.

6. Erlingsson, C. and P. Brysiewicz, *A hands-on guide to doing content analysis.* Afr J Emerg Med, 2017. **7**(3): p. 93-99.
